# Health Benefits, Pharmacological Effects, Molecular Mechanisms, and Therapeutic Potential of α-Bisabolol

**DOI:** 10.3390/nu14071370

**Published:** 2022-03-25

**Authors:** Lujain Bader Eddin, Niraj Kumar Jha, Sameer N. Goyal, Yogeeta O. Agrawal, Sandeep B. Subramanya, Salim M. A. Bastaki, Shreesh Ojha

**Affiliations:** 1 Department of Pharmacology and Therapeutics, College of Medicine and Health Sciences, United Arab Emirates University, Al Ain P.O. Box 15551, United Arab Emirates; 201970113@uaeu.ac.ae (L.B.E.); sbastaki@uaeu.ac.ae (S.M.A.B.); 2 Department of Biotechnology, School of Engineering and Technology (SET), Sharda University, Greater Noida 201310, Uttar Pradesh, India; niraj.jha@sharda.ac.in; 3 Shri Vile Parle Kelavani Mandal’s Institute of Pharmacy, Dhule 424001, Maharashtra, India; sameer.goyal@svkm.ac.in (S.N.G.); yogeeta.goyal@svkm.ac.in (Y.O.A.); 4 Department of Physiology, College of Medicine and Health Sciences, United Arab Emirates University, Al Ain P.O. Box 15551, United Arab Emirates; sandeep.bs@uaeu.ac.ae; 5 Zayed Bin Sultan Center for Health Sciences, United Arab Emirates University, Al Ain P.O. Box 15551, United Arab Emirates

**Keywords:** α-Bisabolol, German chamomile tea, natural products, phytochemicals, sesquiterpene, pharmacology

## Abstract

α-Bisabolol is one of the important monocyclic sesquiterpenes, derived naturally from essential oils of many edible and ornamental plants. It was first obtained from *Matricaria chamomilla*, commonly known as chamomile or German chamomile. The available literature indicates that this plant along with other α-Bisabolol containing plants is popularly used in traditional medicine for potential health benefits and general wellbeing. Nutritional studies are indicative of the health benefits of α-Bisabolol. Numerous experimental studies demonstrated pharmacological properties of α-Bisabolol including anticancer, antinociceptive, neuroprotective, cardioprotective, and antimicrobial. This review aims to collectively present different pharmacological activities based on both in vitro and in vivo studies. In the present review using synoptic tables and figures, we comprehensively present that α-Bisabolol possesses therapeutic and protective activities, therefore, it can be used for potential health benefits based on pharmacological effects, underlying molecular mechanism, and favorable pharmaceutical properties. Based on the studies mostly performed on cell lines or animal models, it is evident that α-Bisabolol may be a promising nutraceutical and phytomedicine to target aberrant biological mechanisms which result in altered physiological processes and various ailments. Given the polypharmacological effects and pleiotropic properties, along with favorable pharmacokinetics, and dietary availability and safety, α-Bisabolol can be used as a dietary agent, nutraceutical or phytopharmaceutical agent or as an adjuvant with currently available modern medicines. The regulatory approval of this molecule for use as food additives, and in cosmetics and fragrance industry is also supportive of its human usage. Moreover, further studies are necessary to address pharmaceutical, pharmacological, and toxicological aspects before clinical or nutritional usage in humans. The biological actions and health benefits open opportunities for pharmaceutical development with pharmacological basis of its use in future therapeutics.

## 1. Introduction

Sesquiterpenes, a subclass of terpenes has attracted a significant interest due to their wide range of biological properties that have been employed in pharmacological and therapeutic research applications. The high identification of sesquiterpenes compounds and their structural variability make this family more distinguishable compared with other plant sourced products. These phytochemicals occupy a large portion of the volatile fraction of aromatic plants. For example, α-bisabolene is a primary component in black pepper (*Piper nigrum*) and β-Caryophyllene in ylang ylang (*Cananga odorata*) [1].

Sesquiterpenes have been known for their wide range of biological functions including anti-infective, antioxidant, anti-inflammatory and anticancer activities and can be obtained from *Matricaria* genus. *Matricaria chamomilla* genus is an herbaceous plant, cultivated in many countries for commercial, pharmaceutical, and cosmeceutical purposes. It belongs to the family of flowering plants, *Asteraceae.* The flowers of *Matricaria chamomilla* have aromatic, coloring and flavoring properties which are implemented in several commercial products. The phytochemical composition of this genus varies and includes volatile sesquiterpene, flavonoid, polyacetylene, and coumarin. *Matricaria chamomilla* has been used anciently in folk medicine as a plant-based remedy for many ailments which survived until today [2]. The key and predominant constituent is α-Bisabolol, where it can be exuberantly found in the plant’s flower buds.

α-Bisabolol also known as levomenol is a monocyclic sesquiterpene alcohol which was first identified and extracted from *Matricaria chamomilla* [3]. However, α-Bisabolol is also present abundantly in various medicinal plants essential oils. The percentage occurrence of α-Bisabolol in numerous plants are listed in Table 1. It exists in two configurations α and β. It’s a clear colorless liquid; with fruity nutty aroma resembling coconut. α-Bisabolol possess favorable physicochemical properties and the characteristics are presented in Table 2. Most of the biological actions are attributed to the α form, on which extensive studies have been conducted. α-Bisabolol has been used as a skin conditioning agent where it is integrated in many cosmetic formulations due to its skin soothing effects, well documented dermal absorption and the absence of dermal irritations or photosensitivity following its application.

The United States Food and Drug Administration (USFDA) has considered α-Bisabolol as a safe compound due to its low toxicity [34]. The effects of α-Bisabolol have been studied in different cell and animal models which indicated its promising beneficial actions. α-Bisabolol demonstrated neuro-, cardio- and nephro-protective, analgesic, anticancer as well as antimicrobial effects offering an intriguing line of evidence for its various therapeutic effects. It has also shown an appreciable chemopreventive activity rendering a great opportunity for further investigations and possible application in chemoprevention. It is thus noteworthy to state that more biological activities and biochemical modifications are likely to emanate from future studies targeting this sesquiterpene compound.

The present review comprehensively summarizes all available studies and aims to advance the understanding regarding the effectiveness of α-Bisabolol in treating different diseases. The broad range of pharmacological properties of α-Bisabolol offers the possibility of incorporating it into various medical formulations.

## 2. α-Bisabolol and Skin Disorders

Skin diseases are among the common illnesses affecting humans at all ages and imposing a huge burden on the global health system [35]. Skin is the body’s largest organ, and it is the first line of defense against a large variety of stressors. Continuous stress can overwhelm defending dermal systems resulting in different cutaneous disorders. Phytochemicals have shown myriad of health effects in curtailing skin damage being used either as individual component or as amalgamated constituents = based formulations [36].

A randomized controlled study revealed that the topical application of a spray containing ozonated sunflower oil and α-Bisabolol achieved a complete healing for venous leg ulcers compared to the control group in which no complete healing was observed [37]. Moreover, the effectiveness of α-Bisabolol was evaluated against atopic dermatitis in a three-center assessor blinded trial on children. Results concluded that α-Bisabolol based cream mitigated eczematous flares compared to baseline showed by a significant decrease in the affected skin surface area, improvement in the total eczema severity score outcome as well as the single area eczema severity score which includes evaluating the infiltration, lichenification and scratching lesions [38].

α-Bisabolol was also found to enhance the effectiveness in attenuating pruritus and inflamed skin in patients with atopic dermatitis when combined with heparin in a topically applied formulation. Furthermore, an improvement was observed in the SCORing Atopic Dermatitis (SCORAD) values in patients who applied the combined therapy as compared with control and individualized therapy groups [39]. On the other hand, the integration of α-Bisabolol in a formulation intended for the treatment of melasma has shown an enhancement in the recovered melasmatic area measured by imaging techniques at the end of treatment duration compared with baseline. Obtained results reported a statistical significance in patients’ satisfaction in terms of improved facial texture, skin oiliness, brightness, hydrated skin, and overall appearance following the treatment [40].

A clinical investigation reported that the use of topical formulation containing α-Bisabolol improved the inflammatory status and showed a reduction in the severity of eczema in treated children [41]. Studies that investigated the potential therapeutic effects of α-Bisabolol in different dermatological disorders are briefly stated in Table 3.

Wound healing is an essential recovery process that maintains skin integrity following a mechanical insult. It is a complex and highly regulated mechanism that consists of inflammatory, proliferation and remodeling phases working on restoring the barrier function of skin. The success of this process is dependent on the intricate interplay among different factors, any aberration which can hinder the normal healing and result in chronic discomfort [42].

**Figure 1:**
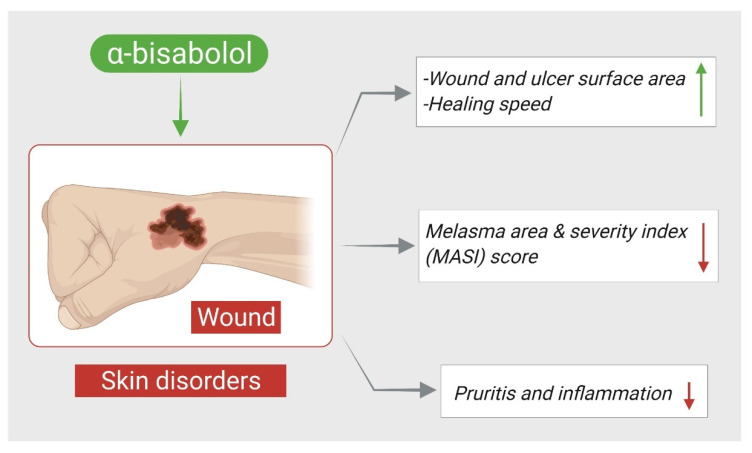
Effects of α-Bisabolol on skin disorders. ↑; increase, ↓; decrease.

α-Bisabolol has been demonstrated to exert a cicatrizant effect on wounds with an ED_50_ of 155 µg/mL. The cicatrization effect was evaluated on mice, where an incision was made, stitched and treatment was applied immediately. This effect is speculated to be attributed to the 1-methylcyclohexene and tertiary hydroxyl structural moieties. An unremarkable action of α-Bisabolol was observed on fibroblasts migration that can hasten the healing process. The study compared the efficacy of α-Bisabolol and the cytotoxicity on BALB/c 3T3 cells proliferation with that of taspine; a potent cicatrizant. It was found that although α-Bisabolol is a less potent compound, but it notably less cytotoxic than taspine [43]. Figure 1 represents the effects of α-Bisabolol on skin disorders and wound healing.

## 3. α-Bisabolol and Neuroprotection

Neuroprotection refers to the integrated strategies that aim to preserve neuronal integrity and health against various insults including genetic mutations and environmental toxins to maintain neuronal function. There are many pathological mechanisms that can predispose the occurrence of neurodegenerative diseases; requiring neuroprotection. Neurodegenerative diseases are a subset of neurological disorders affecting primarily neurons [44].

Parkinson’s disease (PD) is a progressive neurodegenerative disorder with neuropathological features of degraded dopaminergic neurons and accumulated α-synuclein depositions. Pharmacological treatment of PD is acquiring increased attention due to the challenges researchers facing during the quest of novel therapeutic approaches [45]. The neuroprotective effect of α-Bisabolol was first described on *Drosophila melanogaster* model of rotenone induced toxicity [46]. The study reported an improvement in locomotor activity, a reduction in the expression of thiol and a reinstate of the activity of mitochondrial complex-I. α-Bisabolol also increased the mRNA level of antioxidants proteins such as superoxide dismutase (SOD), catalase (CAT), and the keap1 gene product. A more recent study investigated the neuroprotective role of α-Bisabolol against rotenone induced neurodegeneration in a rat model of PD. Results showed a promising effective action of α-Bisabolol in abrogating neuronal degradation associated with PD in both Substantia Nigra and Striatum. α-Bisabolol attenuated oxidative insult by reducing malondialdehyde (MDA), restoring depleted glutathione (GSH) and improving SOD and CAT activity. It also attenuated neuroinflammation by reducing glial cells activation and subsequent release of proinflammatory cytokines (IL-1β, IL-6 and TNF-α) and mediators (iNOS and COX-2). Additionally, α-Bisabolol ameliorated apoptosis by reversing the downregulated Bcl-2, and the upregulated Bax, cleaved caspases-3 and 9 levels. It was also observed that α-Bisabolol restored mitochondrial function by preventing mitochondrial lipid peroxidation, cytochrome-C release and most importantly preserving Complex-I activity [47].

Another neurodegenerative disease is Alzheimer disease (AD), the pathogenesis of which is known to be attributed to the deposits of β-amyloid (Aβ) peptide affecting neuronal connectivity and synaptic function resulting in impaired cognitive function [48]. Therefore, the inhibition of Aβ aggregation has been considered the cornerstone in developing disease modifying agents for AD. Using Aβ_25–35_ induced neurotoxicity in neuro2a cells and transgenic *Caenorhabditis elegans*, α-Bisabolol was evaluated for its efficacy in protecting against the neuronal insult. Cellular viability and morphology were preserved following α-Bisabolol treatment. As proposed by many hypotheses that reactive oxygen species (ROS) and reactive nitrogen species can trigger Aβ accumulation. Therefore, the level of ROS and nitrite were measured to explore the protective mechanisms of α-Bisabolol. The study concluded that α-Bisabolol safeguarded against the induced upsurge of ROS and nitrite. Lipid and protein peroxidation is considered the interconnected result of ROS accumulation. For further confirmation, finding showed that protein carbonyl content (PCC) was reduced in α-Bisabolol treated cells compared to untreated group. α-Bisabolol treatment also restored mitochondrial membrane potential (MMP) validating its antioxidant effect. Acetylcholinesterase (AChE) and butylcholinesterase are enzymes that control the degradation of Acetylcholine (Ach) and its subsequent abundance in synaptic cleft. Substantial evidence implicates that the inhibition of AChE forms a curative approach in AD patients. Therefore, the inhibitory activity of α-Bisabolol was evaluated in N2a cells and showed a significant reduction in AChE activity and an ability to avert Ach depletion. At the same time, α-Bisabolol protected cells from Aβ triggered apoptosis by reducing Bax and Caspase-3 and increasing Bcl-2 activity. Confocal laser scanning microscope also detected more viable cells with intact morphology than apoptotic cells in α-Bisabolol group. BACE1 (β-secretase βAPP-cleaving enzyme 1) is another enzyme that requires attention due to its participation in Aβ release.

Same study evaluated α-Bisabolol inhibitory activity on BACE1 and found a decrease in BACE1 activity following α-Bisabolol treatment. α-Bisabolol also preserved the viability of *Caenorhabditis elegans* by increasing their life span [49]. These findings were previously confirmed by another study evaluating α-Bisabolol effect on Aβ aggregation and survival of PC12 cells following exposure to Aβ due to the anti-aggregation property of α-Bisabolol against Aβ peptide. It also decreased apoptotic cell death and increased the cellular progenicity [50].

**Figure 2:**
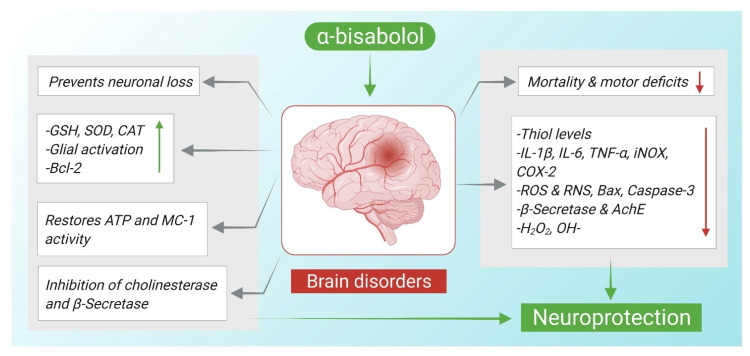
Neuroprotective mechanisms of α-Bisabolol against neurodegeneration. ↑; increase, ↓; decrease.

Recently, α-Bisabolol has been subjected for an additional assessment for acquiring corroborating evidence regarding its efficiency in combating AD associated pathology. α-Bisabolol was tested for its antioxidant, anti-aggregate, anti-AchE and anti-apoptotic activities. Neuro-2a cells treated with α-Bisabolol had better ROS and RNS scavenging potential, decreased MDA and protein carbonyl level, restored MMP loss and prevented induced apoptosis. Moreover, AChE, BuChE, β-secretase actions were decreased significantly in cells pretreated with α-Bisabolol [51]. On the other hand, a synthesized compound of α-Bisabolol; α-Bisabolol β-D-fucopyranoside (ABFP) was evaluated for its activity against β-amyloid toxicity in both in vivo and in silico experiments. The compound clearly illustrated a potent anti-AchE activity of 95.869% similar to the activity of donepezil, a standard drug. It had also a profound antioxidant potential demonstrated by both hydrogen peroxide and hydroxyl radical scavenging and a metal chelating activity. The compound also disaggregated Aβ_25–35_ peptide and protected against its induced toxicity by increasing neuro2a cells viability [52]. In Figure 2, the mechanisms of α-Bisabolol in mediating neuroprotection are summarized.

Cerebral ischemia is a neurological insult which can lead to irreversible damage or even death if not treated promptly and effectively. The principal in cerebral ischemia treatment is to restore blood flow to the deprived regions by reperfusion. This process can result in cerebral ischemia-reperfusion injury that requires neuroprotection due to the accompanying biochemical disturbances occurring in brain tissue [53]. In a model of permanent occlusion of the middle cerebral artery (pMCAO) inducing cerebral ischemia in mice, α-Bisabolol was evaluated for its neuroprotective effects. It was observed that there was a decrease in cerebral infarct area, a prevention of memory deficits and an improvement in motor performance in mice treated with α-Bisabolol following ischaemia. In addition, the cresyl-positive cells (viable cells) were increased by α-Bisabolol treatment compared with untreated mice. However, the counted Fluoro-Jade C positive cells (dead cells) were reduced in the group of mice treated with α-Bisabolol; indicating the inhibitory effect of α-Bisabolol on cellular degradation. At the same time, the induction of proinflammatory markers expression after pMCAO was suppressed by α-Bisabolol administration, where the expressions of myeloperoxidase (MPO), TNF-α, iNOS and the astroglial marker, GFAP were reduced in α-Bisabolol treated mice [54]. The neuroprotective effects and demonstrated pharmacological mechanisms of α-Bisabolol are presented in Table 4.

## 4. α-Bisabolol and Anticancer Effects

**Figure 3:**
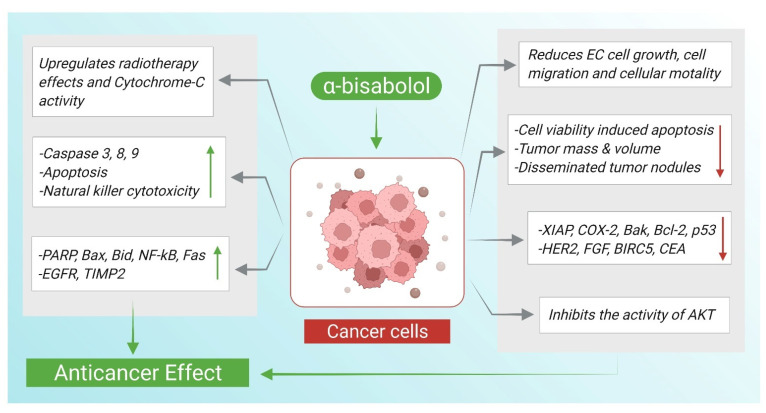
α-Bisabolol mediated anticancer actions and demonstrated mechanisms. ↑; increase, ↓; decrease.

Cancer is characterized by cellular overgrowth with abnormal proliferation which can metastasize to surrounding tissues. Cancerous cells have dysregulated cell division cycle lacking the tight control required for regulated cell replication resulting in an altered mitotic activity. In addition to the uncontrolled growth, cancerous cells can easily evade apoptosis due to mutated genes responsible for apoptosis control [55]. Apoptosis plays an essential role in eliminating abundant cells and terminating continuous cell growth. Targeting apoptosis has been shown to be an effective strategy in treating cancer demonstrated by many anticancer drugs stimulating pro-apoptotic proteins and inhibiting anti-apoptotic ones [56]. The anticancer actions and underlying mechanisms of α-Bisabolol are illustrated in Figure 3.

In order to envisage the exact apoptotic mechanism of α-Bisabolol, a group of researchers hypothesized that α-Bisabolol can be embedded in the lipid rafts easily, which are known to be abundant in cancerous cells. At the same time, Bid; a member of the Bcl-2 pro-apoptotic family proteins, has been reported to be easily recruited in the lipid rafts by apoptosis-inducing agents, conferring it a role in the formation of a death-inducing signaling complex. With the aid of Gas Chromatography Mass Spectroscopy (GC-MS) which measured α-Bisabolol amount in the cell extracts, it is revealed that α-Bisabolol was adsorbed into flottillin rich structures known as the lipid rafts. Bid was also shown by western blotting to be present in the flottillin-enriched fraction, referring to α-Bisabolol induced movement of Bid to lipid rafts-rich membrane regions. The Surface Plasmon Resonance (SPR) analysis also indicated that α-Bisabolol interacts directly with Bid. Thus, it can be inferred that α-Bisabolol apoptotic action is mediated through its interaction with Bid protein [57].

The anticancer effects and mechanisms of α-Bisabolol in numerous types of cancer including, pancreatic, endometrial, breast and liver have been demonstrated in many experimental studies. Endometrial cancer is the main gynecological malignancy occurring in the inner lining of the uterus (endometrium) [58]. The anticancer activity of α-Bisabolol was investigated on endometrial cancer (EC) cell line on which α-Bisabolol exhibited an antitumor action by decreasing cellular viability, migration, and invasion ability of EC cells. α-Bisabolol was shown to promote apoptosis indicated by significantly increasing caspase-3 and correspondingly decreasing its direct inhibitor; X-linked inhibitor of apoptosis protein (XIAP). Cyclooxygenase-2 (COX-2) and Poly ADP-ribose polymerase (PARP) are considered as downstream mediators of caspase-3 effects. Therefore, their levels were further assessed revealing a reduced COX-2 and an increased cleaved PARP following α-Bisabolol treatment. There was also a synergistic improvement in the inhibitory outcome on EC cell growth when α-Bisabolol was combined with radiotherapy along with radiosensitivity enhancement[59].

The role of α-Bisabolol in glioblastoma, a tumor originating from glial cells of the central nervous system is well studied. Glioblastoma is considered among the most intimidating and aggressive types of tumors due to its poor prognosis and deleterious repercussions [60]. α-Bisabolol was evaluated for its cytotoxic effect against U138-MG human and C6 rat glioma cells. A3 is one of adenosine receptor subtypes on which is known as a cell death mediator in glioma. α-Bisabolol treated cells showed reduced viability and increased activity of ecto-5′-nucleotidase (ecto5′-NT/CD73); an enzymatic source for adenosine production. Since ecto-5′-NT/CD73 stimulation produces high levels of adenosine which might possibly be involved in inducing cell death through A3 adenosine receptor, the participation of this receptor was also investigated. Hence, C6 cells were pretreated with MRS1220, an A3 receptor antagonist. α-Bisabolol pretreatment significantly reversed the decrease in the number of the viable cells. An indication of the involvement of adenosinergic system represented by ecto-5’-nucleotidase/CD73 and A_3_ receptor in the anti-proliferative action of α-Bisabolol on glioma cell lines [61]. Similar findings were reported by an earlier study which found that α-Bisabolol induced a cytotoxic effect on glioma cells with an inhibitory effect of 50% compared to untreated cells. The study also found that α-Bisabolol can mediate apoptosis by inducing cytochrome-C translocations from mitochondrial membrane [62].

Hepatocellular carcinoma is a malignancy affecting the liver with a growing tumor by increased number of cells resembling hepatocytes affecting patients with chronic liver diseases and cirrhosis [63]. The apoptosis inducing effect of α-Bisabolol was investigated on a model of human liver carcinoma cell line HepG2. It was observed that cleaved caspases 3, 8 and 9 were of a higher concentration in the α-Bisabolol treated cells as compared with untreated. In addition, α-Bisabolol decreased the level of mitochondrial cytochrome-C, increased the cytosolic cytochrome-C content along with an up-regulation in the pro-apoptotic proteins; Bax and Bid and a down-regulation of anti-apoptotic Bak and Bcl-2 proteins. The expression of p53 (a transcription factors whose products might lead to apoptosis), NF-κB and Fas was increased following α-Bisabolol treatment, indicating their function in mediating α-Bisabolol-induced apoptosis in the cancer cell line. At the same time, MTT assay showed the effectiveness of α-Bisabolol in inducing cytotoxicity in HepG2 cells [64].

Another study provided additional evidence for the anticancer potential of α-Bisabolol by examining it on non-small cell lung cancer (NSLC) cell line. Lung cancer is histologically subdivided into non-small cell lung cancer; a cancer type that is associated with major cases of cancer related mortality, and small cell lung cancer [65]. In efforts to elucidate the underlying mechanisms of α-Bisabolol anticancer effect, the study found that α-Bisabolol decreased p-PI3K and p-AKT activation; a signaling pathway, the activation of which is known to play a role in cancer pathogenesis. It was also demonstrated that α-Bisabolol suppresses the cellular proliferation at G2/M cell cycle phase. Bax was increased and Bcl-2 was decreased in α-Bisabolol-treated cells [66].

In pancreatic cancer, an aggressive adenocarcinoma with perineural and vascular growth rendering it resistant to surgical and conventional interventions [67], AKT phosphorylation was suppressed by α-Bisabolol when tested on pancreatic cancer cell line. It is speculated that AKT activation can suppress the activity of EGR1; a tumor suppressor molecule [68]. Consequently, the α-Bisabolol antitumor effect might be attributed to the activation of EGR1. The expression of EGR1 was highly elevated following the treatment with α-Bisabolol. TUNEL staining further confirmed the anti-apoptotic effect of α-Bisabolol evident by the high number of TUNEL-positive cells after pancreatic cancer cells being treated with α-Bisabolol [69]. 

Furthermore, α-Bisabolol was shown to inhibit the invasiveness of pancreatic cancer cell lines including KLM1, KP4 and Panc1. In looking into the key modulators of this effect, Polymerase chain reaction (PCR) analysis revealed an upregulated level in α-Bisabolol-treated cells for each of Kisspeptin-1 receptor (KISS1R), metastasis suppressor 1 (MTSS1) and tissue inhibitor of metalloproteinase 2 (TIMP2). To ensure the participation of KISS1R and its ligand KISS1 (metastasis suppressors) in the invasiveness inhibitory effect of α-Bisabolol, KISS1R was suppressed in the pancreatic cancer cells. This suppression was correlated with an attenuation of the inhibitory effect of α-Bisabolol on cancer cells migration [70].

Acute leukemia is a malignant disorder of blood constituting organs in a way where healthy bone marrow is replaced by undifferentiated cells [71]. Cells of acute leukemia were treated with α-Bisabolol and found sensitive to α-Bisabolol-induced growth inhibitory effect [72]. α-Bisabolol has been additionally scrutinized for its role against the carcinogenesis of breast cancer, a frequently diagnosed malignancy with the highest incidence in women. It was found that the treatment with α-Bisabolol reduced the incidence of tumor masses as shown by increased percent of tumor free mice and delayed appearance of tumors in HER2/neu transgenic mice. It is also reported that α-Bisabolol boosted the immunity response by T-cell subsets (CD4 and CD8) supplementation in treated mice. Gene expression profiling detected downregulated Spp1, Birc5 (carcinogenesis genes) and Egfr, Fgf1 (angiogenesis genes), in addition to a significant downregulation in neu gene (Erbb2) following α-Bisabolol administration [73].

Moreover, α-Bisabolol derivative demonstrated a potent anti-proliferative effect on pancreatic cancer cells illustrated by the increased percentage of total apoptotic cells. α-Bisabolol also showed a growth inhibitory effect, but comparatively less that its derivative. The derivative also reduced the volume of xenograft tumor along with peritoneal metastasis, changed cell morphology with the loss of membrane asymmetry indicative for apoptosis induction, suppressed AKT expression and reduced the tumor makers CEA and CA19-9 [74].

More analogues were synthesized by glycosidation of α-Bisabolol which proved efficient cytotoxicity in vitro on human and rat glioma cells. α-Bisabolol α-L-rhamnopyranoside showed the most potent activity with IC50 reaching up to 64 M. Accordingly, it was concluded that the addition of a sugar moiety has amplified the cytotoxic effect of α-Bisabolol giving an emphasis on the importance of structural modifications [75]. In the same context, thiosemicarbazones; derived from α-Bisabolol were tested for their antitumor effect against human tumor cell lines. The synthesized derivatives were effective in inhibiting the growth of cells with the ketone analogue reported as the most potent [76]. The studies pertaining to the anticancer actions of α-Bisabolol are summarized in Table 5.

## 5. α-Bisabolol and Antinociception

Nociception is a sensory mechanism of encoding noxious stimuli and perceiving them as pain. The process is transduced by nociceptors that can detect damaging sensory input and translate it as a painful stimulant [77]. α-Bisabolol has been speculated as a promising anti-nociceptive agent, where its analgesic effect and the corresponding implicated mechanisms have been subjected to an extensive investigation in different pain models. In an infraorbital nerve transection (IONX) induced nociception and trigeminal central sensitization in rats, α-Bisabolol administration was associated with a significant reversal of decreased threshold of mechanical sensitization in both ipsilateral and contralateral sides of the rat face as well as decreasing IONX-induced medullary dorsal horn (MDH) nociceptive neurons sensitization [78].

In a model of chronic pain, it is defined as a prolonged persistent pain that can be originated from inflammatory or neuropathic stimuli, α-Bisabolol was evaluated in reducing inflammatory and neuropathic associated pain induced by Freund’s Complete Adjuvant (FCA) and partial lesion of the sciatic nerve (PLSN), respectively. When assessing mechanical hyperplasia, α-Bisabolol treated mice showed an increase in paw withdrawal threshold in hyperplasia induced by injected CFA and the partial sciatic nerve injury. α-Bisabolol also caused an anti-allodynic effect following acetone application on mice paw with lesioned nerve and it reduced gliosis evident by decreased immunostaining of ionized calcium-binding adapter protein (Iba-1) as well as decreased TNF-α production along with IL-10 release [79]. Moreover, the anti-nociceptive activity of α-Bisabolol was also assessed on formalin, capsaicin and glutamate induced orofacial pain in mice. α-Bisabolol administration resulted in decreased face-rubbing behavior in mice compared with control mice. This effect was suggested due to the observed inhibitory effect of α-Bisabolol on TNF-α production measured in pleural inflammatory exudate following carrageenan induced pleurisy [4].

**Figure 4:**
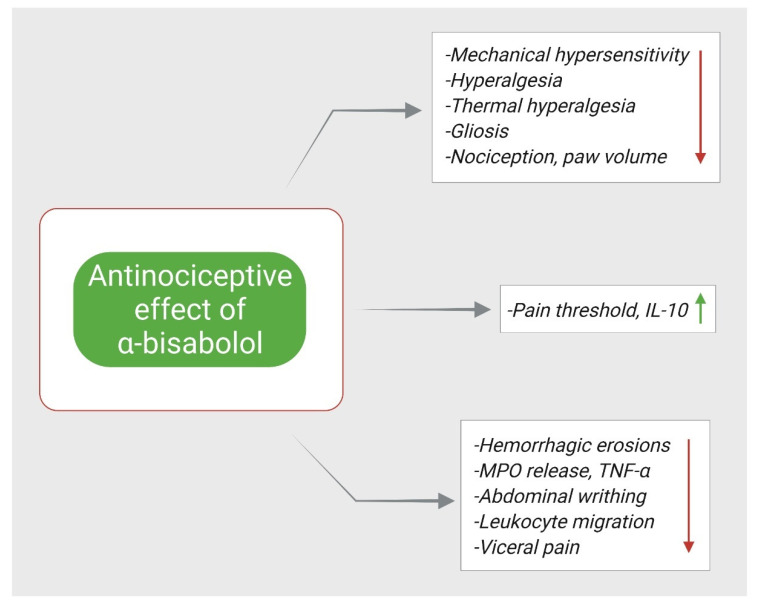
Antinociceptive mechanisms of α-Bisabolol. ↑; increase, ↓; decrease.

In the same context, a study aimed to investigate the mechanism involved in α-Bisabolol attenuation of orofacial pain. The study found that the administration of HC-030031; a TRPA1 antagonist, enhanced α-Bisabolol efficacy in reducing orofacial pain induced by formalin, whereas the administration of naloxone (opioid antagonist), L-NAME (nitric oxide antagonist), and glibenclamide (ATP-sensitive K^+^ channel (K^+^ ATP blocker), did not alter the efficacy of α-Bisabolol. Furthermore, in cinnamaldehyde (a TRPA1 agonist) induced pain, HC-030031 did not result in an enhancement in anti-nociception of α-Bisabolol. Hence, it can be speculated the α-Bisabolol anti-nociceptive effect is due to TRPA1 receptor blockage [80]. Similar finding was reported by different study, where they found that the administration of ruthenium red (a non-competitive antagonist of TRPV1) led to additive anti-nociceptive effects with α-Bisabolol [81]. Figure 4 demonstrates the mechanisms by which α- Bisabolol exerts its anti-nociceptive effects.

To elucidate the possible involved mechanism behind α-Bisabolol anti-nociception, a conducted study observed that α-Bisabolol inhibited nerve excitability of mice peripheral nervous system in a manner similar to lidocaine, suggesting a probable blockage of voltage dependent sodium channels [82]. In addition, it was observed that mice pretreated with α-Bisabolol displayed a reduction in paw-licking time following formalin injection and abdominal writhing induced by acetic acid. This is in consistent with a remarkable reduction in leukocytes migration, myeloperoxidase (MPO) activity, TNF-α in the peritoneal fluid of rats with peritonitis and decreasing edema volume induced by carrageenan, dextran, and 5-HT but not histamine [83]. In test models of visceral pain induced by cyclophosphamide and mustard oil, α-Bisabolol was evaluated and showed an ability to reduce pain related behavior in treated mice [84]. Similarly, corneal nociception was notably reduced, inferred from the decreased eye wipes by mice forepaws, on which α-Bisabolol containing ointment was applied [85]. Furthermore, it was reported that α-Bisabolol had a synergistic effect in reducing nociception, inflammation and gastric injury when co-administered with diclofenac [86].

Another aspect pertaining to α-Bisabolol efficiency enhancement was carried out to ensure more efficient anti-nociception effect. Nanocapsules containing α-Bisabolol were applied topically to mice eyes and provided a relief from triggered nociception [87]. Clinically, α-Bisabolol containing mouthwash was evaluated on thirty patients undergoing maxillofacial surgery in a randomized controlled trial. The study found similar efficiency of α-Bisabolol based mouthwash to that of chlorhexidine in reducing pain during brushing and the urge of rubbing or wiping sutures and lesions [88]. Collectively, this data (briefly summarized in Table 6) suggests that α-Bisabolol possess an anti-nociceptive effect that may led development of α-Bisabolol as a future analgesic agent to relieve pain of various origins.

## 6. α-Bisabolol and Cardioprotection

Cardioprotection implies all interventions meant for preventing, ameliorating and repairing myocardial injuries following myocardial insults. It has been of great importance for research to develop pharmacological and mechanical interventions that induce cardioprotection. There are many injurers that necessitate the application of critical cardioprotection [89]. Myocardial infarction (MI) is an ischemic injury of the heart arises due to a blockage in arteries supplying heart with blood and oxygen leading to heart deprivation from these two nourishing elements [90].

To elucidate the underlying cellular perturbations in MI to develop effective therapeutic agents, α-Bisabolol was investigated for its protective properties in MI induced by isoproterenol in rats. The study has provided substantial evidence on the vital role α-Bisabolol has in ameliorating MI damage. α-Bisabolol was found to be capable of decreasing the level of serum lactate dehydrogenase (LDH), lipid peroxidation reflected by decreased amount of thiobarbituric acid reactive substances (TBARS) as well as myocardial infarct size. α-Bisabolol also exhibited an antioxidant effect manifested by elevating the activity of both SOD and CAT.

Lysosomal dysfunction was attenuated in α-Bisabolol receiving rats, where the level of lysosomal enzymes including β-glucuronidase, β-galactosidase, cathepsin-B and cathepsin-D were reduced as well as their leakage into cytosol was prevented. Furthermore, the study reported a decrease in TNF-α, IL-6, IL-1β levels and iNOS and COX-2 activities, concurrently with increased IL-10. The anti-inflammatory potential also included a NLRP3 inflammasome activation inhibition by suppressing TLR4 mediated NF-κB/MAPK pathway evidenced by measured (TLR4, p-NFκB-p65, p-IκBα), MAPK (p-ERK1/2, p-P38, p-JNK) and inflammasome complexes (NLRP3, ASC, pro-IL-1β, caspase-1-p20 and TXNIP) proteins in heart. Furthermore, an autophagy modulation was observed, where autophagic proteins (Beclin-1, LC3BI/II,) were increased, p-mTOR was decreased along with active formed autophagic vacuoles visualized under the electron microscope in α-Bisabolol treated rats compared with isoproterenol. The findings were further supported by the preservation of histological architecture of myocardium ascribed to the lessened inflammatory cells and reduced separation of cardiac muscle fibers [91].

The authors further investigated in a preceding study the effects of α-Bisabolol on mitochondrial dysfunction and apoptosis in MI induced by isoproterenol. The results proved that α-Bisabolol can protect against triggered mitochondrial dysfunction and apoptosis in MI. The mitochondrial lipid peroxidation was increased in isoproterenol injected rats shown by high levels of lipid hydroperoxide (LOOH) and TBARS, with decreased SOD, CAT activities and GSH level. However, rats co-treated with α-Bisabolol showed reduced LOOH and TBARS and increased SOD, CAT and GSH. α-Bisabolol also decreased Ca^2+^ overload, mitochondrial swelling, and increased ATP production. The activities of Krebs cycle dehydrogenases (isocitrate dehydrogenase (ICDH), succinate dehydrogenase (SDH), malate dehydrogenase (MDH), α-ketoglutarate dehydrogenase (α-KGDH)) and inner mitochondrial electron transport chains (ETC) complexes (I, II, III and IV) were maintained by α-Bisabolol administration.

**Figure 5:**
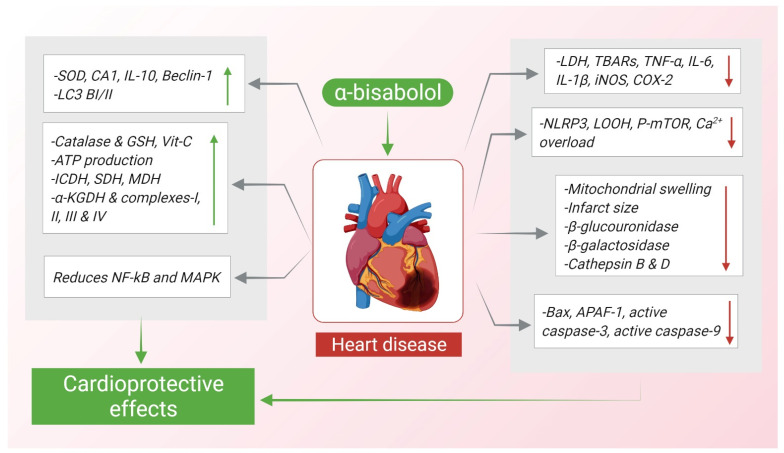
Schematic summary of cardioprotective actions of α-Bisabolol. ↑; increase, ↓; decrease.

Furthermore, creatine kinase (CK) and LDH were increased and normal architecture of mitochondria with intact myofibrils were preserved by α-Bisabolol. Apoptosis induced by isoproterenol was inhibited by α-Bisabolol which downregulated Bax, P53, APAF-1, active caspase-3, active caspase-9, inhibited cytochrome-C release and upregulated the expression of Bcl-2 protein [92]. Figure 5 shows how α-Bisabolol protects the heart against myocardial insults.

α-Bisabolol is also able to reverse the increase in heart rate (HR), systolic (SBP), diastolic (DBP), and mean arterial pressure induced in MI and to maintain them within normal range [93]. An in silico based investigation by docking detected an ability of α-Bisabolol to interact with receptors implicated in cardiovascular related health including α-glucosidase, angiotensin- converting enzymes, beta-2 adrenergic receptor, glucocorticoid, HMG-CoA reductase, insulin, mineralocorticoid, potassium channel receptors and peroxisome proliferator-activated receptor alpha, suggesting its likeliness to be a cardiovascular diseases modifying agent based on it its ability to interact on these receptors [94]. The cardioprotective effects and demonstrated mechanisms of α-Bisabolol are summarized in Table 7.

## 7. α-Bisabolol and Antimicrobial Effects

Antimicrobials are drugs with an ability to inhibit or kill the pathogenic microorganisms that can cause infectious diseases which in turn can be a cause of morbidity and mortality [95]. The emergence of micro-organisms resistant to conventional antimicrobials with the concept of multiple drug resistance has urged scientists to look for alternative antimicrobial substances from various sources especially plant derived origin due to their abundance, biodiversity, and low side effects [96].

The antimicrobial activity of α-Bisabolol was evaluated against *Escherichia coli*, *Staphylococcus aureus*, *Candida albicans*, *Candida krusei*, *Candida tropicalis*, and multi-resistant bacterial strains, *Staphylococcus aureus* and *Escherichia coli*. Results showed that all strain were sensitive to α-Bisabolol which also exerted a synergism when applied with aminoglycosides [5]. In the same manner, α-Bisabolol demonstrated an antibacterial effect against *Staphylococcus aureus*, *Escherichia coli* and *Pseudomonas aeruginosa* as well as a synergism against *S. aureus*, when combined with the antibiotic norfloxacin and against *E. coli* when combined with gentamicin [97]. In digging into the antibacterial mechanisms associated with α-Bisabolol, α-Bisabolol was tested for a potential inhibition of TetK and NorA efflux pump responsible for pumping out antibiotics into extracellular space and thus rendering the bacteria resistant to the administered antibiotic. The combination of α-Bisabolol with tetracycline and norfloxacin resulted in potentiation of their action, illustrated by a reduction in their minimum inhibitory concentration (MIC) from 192 µg/mL to 128 µg/mL against SA IS-58 TetK pump expressing strain and from 256 μg/mL to 32 μg/mL against SA 1199 B NorA pump expressing strain, respectively [98].

Furthermore, α-Bisabolol exerted a concentration-dependent effect on decreasing the number of viable colonies of *Bacillus Solobacterium Moorei* associated with halitosis. The bacterial cell wall presented a concurrent enhanced permeability of antimicrobials such as tea tree oil after being exposed to α-Bisabolol, making it an effective agent in oral hygiene products for eliminating microbes from oral cavity and reducing halitosis [99]. *Staphylococcus aureus* and *Escherichia coli* were shown to be more sensitive to the effects of each of ciprofloxacin, clindamycin, erythromycin, gentamicin, tetracycline, and vancomycin when applied in concomitantly with α-Bisabolol [100]. This suggests the practical utility of α-Bisabolol in increasing the susceptibility of bacteria against antibiotics. The studies demonstrated anti-microbial potential of α-Bisabolol are presented in Table 8.

Fungal infections are considered predominant infectious diseases with a prominent resistance to commercially available synthetic antifungals which are associated with serious side effect such as hepatotoxicity caused by azole group [101]. Therefore, it is becoming of great importance to find compounds of plant origin with potent antifungal activity and safe to administer.

The antifungal activity of α-Bisabolol was evaluated on *Aspergillus fumigatus* species. Results demonstrated that α-Bisabolol is capable of inhibiting the growth of *A. fumigatus* as well as inhibiting the synthesis of ergosterol responsible for cellular membrane integrity. The expression of erg6 gene encoding for ∆^24^-sterol methyltransferase (24-SMT); an enzyme responsible for converting lanosterol to eburicol an integral contributor in ergosterol synthesis, was suppressed in *A. fumigatus* treated with α-Bisabolol. The activity of 24-SMT was also assayed for further assertion. The assay revealed a notable decrease in its functionality observable by the reduction in the transmethylation activity [102]. On nine different species of dermatophytes, α-Bisabolol along with other natural compounds were evaluated for their antidermatophytic activity. α-Bisabolol was superior to all other compounds in inhibiting the growth of fungal species, where it exerted over than 50% growth inhibition values on tested fungi. It is noteworthy that α-Bisabolol had an IC_50_ value lower than that of fluconazole against some fungal species.

*Microsporum gypseum* which displayed a particular sensitivity for α-Bisabolol was used for carrying on further investigations. Resazurin Assay confirmed that α-Bisabolol is good at inhibiting spores’ germination of *M. gypseum* with an increasing efficiency corresponds to an increase in the concentration. At the same time the transmission electron microscopy visualized the anomalies caused in *M. gypseum* organelles including irregular nucleus, vacuolization, and formation of multiple septa [103].

Additionally, α-Bisabolol exhibited a more potent growth inhibition for *Fusarium oxysporum* with low minimum inhibitory concentration (MIC) when compared with NaCl. The combination of both α-Bisabolol and NaCl resulted in an eightfold reduction in NaCl MIC, indicating a favorable synergism aimed to decrease administered antifungal agent [104]. On the other hand, α-Bisabolol was evaluated for an antiparasitic activity on *Trypanosoma cruzi*, where it was indicated that, α-Bisabolol induces cell death in *Trypanosoma cruzi* by triggering apoptosis, increasing ROS production, causing mitochondrial transmembrane potential dysfunction, and inhibiting tcGAPDH enzyme; detected by docking study which revealed that α-Bisabolol occupies the same site as chalepin (known inhibitor of tcGAPDH) does [105].

The biocide action of α-Bisabolol was also investigated on *Leishmania infantum promastigotes;* the main species causing Leishmaniases. α-Bisabolol marked a significant inhibitory effect at all concentrations tested with an inhibition of 100% for the highest one [106]. Another study also agreed that α-Bisabolol possess the highest efficiency in inhibiting *L. infantum* growth with low IC_50_. Obtained results showed that α-Bisabolol-induced apoptosis, led to phosphatidylserine externalization, disrupted plasma membrane permeability, and decreased the potential of mitochondrial membrane as well as the ATP level [107]. A performed evaluation by light microscopy on the ultrastructural changes revealed that α-Bisabolol treated *Leishmania amazonensis promastigotes* and *amastigotes* showed mitochondrial swelling, numerous vacuoles formation, lipid inclusions of different sizes, condensed nucleus, and severely damaged cytoplasm [108].

The cytotoxic activity of α-Bisabolol against *Leishmania tropica promastigotes* was attributed to triggering ROS production, mitochondrial membrane depolarization, phosphatidilserine (PS) translocation, ultrastructural changes, and disrupted amastigotes cells [109] An in vivo study proved that α-Bisabolol is an effective orally administered compound in reducing the parasitic load in mice spleen and liver with no significant adverse effects on mammalian cells [110]. In a clinical and explanatory study, α-Bisabolol efficacy was assessed on canine leishmaniosis naturally infected dogs and compared with meglumine antimoniate as a reference drug. α-Bisabolol was effective in reducing the parasite load in bone marrow, lymph node and peripheral blood, increasing the release of IFNγ and inducing a Th1 cell mediated immunity. These effects were reproducible in most dogs received α-Bisabolol in comparison with the effect of the reference drug which were observed in only one dog out of six receiving it [111].

The topical application of α-Bisabolol in treating cutaneous leishmaniasis was evaluated on hamsters for an antileishmanial potential. The formulation containing α-Bisabolol was observed to be associated with a decrease in the lesion and footpad thickness and with up to 83% reduction in the parasite load in comparison with the reference group [112]. These findings are suggestive of the promising possession of antileishmanial properties of α-Bisabolol in treating visceral leishmaniosis.

**Table 8:** The antimicrobial actions of α-Bisabolol against different bacterial strains.

Antimicrobial Actions				
Compound	Dose/Route/ Duration	Model	Major Mechanisms	Reference
α-Bisabolol	4–512 μg/mL (for bacteria) 3–4096 μg/mL (for fungus)	*Staphylococcus aureus, Candida albicans, Candida krusei, Candida tropicalis*	Inhibited microbial growth	[5]
α-Bisabolol	1024 μg/mL	*Staphylococcus aureus, Escherichia coli* and *Pseudomonas aeruginosa*	Inhibited microbial growth	[97]
α-Bisabolol	1024 µg/mL	*Staphylococcus aureus* strains: pT181 carrying the TetK efflux pump protein that extrudes tetracycline; and the 1199B that presents resistance to norfloxacin by NorA pump expression	↓ MIC for tetracycline and norfloxacin	[98]
α-Bisabolol	0.1%	*Bacillus Solobacterium moorei*	↓ colonies number ↑ effect of tea tree oil	[99]
α-Bisabolol	0.5–2 mM	*Staphylococcus aureus* and *Escherichia coli*	↑ effect of co-administered antibiotics	[100]
α-Bisabolol	0.281–9 mM for 3 days	*Aspergillus fumigatus* Af239	↓ fungal growth (-) 24-SMT, ↓ erg6	[102]
α-Bisabolol	5, 10, 20, 50, 100, 200 μg/mL	*Microsporum gypseum, Microsporum canis, Trichophyton violaceum, Nannizzia cajetani, Trichophyton mentagrophytes*, *Epidermophyton floccosum*, *Arthroderma gypseum, Trichophyton rubrum* and *Trichophyton tonsurans*	↓ fungal growth (-) spore germination ↑ morphological anomalies	[103]
α-Bisabolol	1 µg/mL	*Fusarium oxysporum*	↓ fungal growth ↓ MIC of NaCl	[104]
α-Bisabolol	1000–31.25 μM	*Trypanosoma cruzi Y* infected LLC-MK2 cells	↓ cell viability ↑ ROS, ↑ apoptosis	[105]
α-Bisabolol	1000–6.25 μg/mL	*Leishmania infantum zymodeme 1*	↓ parasite growth	[106]
α-Bisabolol	IC_50_ = 9.5, 16.0	*Promastigotes of Leishmania infantum* and *amazonensis*	↓ parasite growth ↑ apoptosis, ↓ Δψm ↓ ATP, ↑ membrane permeabilization	[107]
α-Bisabolol	1.86–60 μg/mL (IC_50_ = 8.07 μg/mL)	MHOM/BR/76/Ma-76 *Leishmania amazonensis* strains	(-) parasite growth ↑ morphological changes	[108]
α-Bisabolol	50, 200, and 1000 mg/kg p.o for 14 days	10^7^ stationary-phase *promastigotes of Leishmania infantum* injected in mice	(-) parasite growth	[110]
α-Bisabolol	25 and 100 μM IC_50_ = 25.2 μM	*Leishmania tropica promastigotes*	(-) parasite growth ↑ ROS, ↑ apoptosis ↑ ultrastructure changes ↑ PS externalization	[109]
α-Bisabolol	30 mg/kg, p.o, once daily for 28 days	*Canine leishmaniosis* naturally infected dogs	↓ parasite load ↓ antibody titers ↑ IFNγ, ↑ Th1/Th2 immunity	[111]
α-Bisabolol	1%, 2.5%, 5% applied ointment, 200 mg/kg p.o. for 21 days	Inoculated 3 × 10^7^ parasites in the left hind footpad of hamsters	↓ lesion thickness ↓ parasite load	[112]

Symbols indications: ↑; increase, ↓; decrease, (-); reduce.

## 8. α-Bisabolol and Gastroprotection

The core function of stomach is to aid in food digestion which makes the stomach frequently exposed to various noxious factors such as hydrochloric acid, regurgitated bile acid and wide range of osmolality. The resilience of stomach in withstanding several insults is conferred by the physiological responses of gastric mucosa and the ability to self-repair induced gastric lining damages [113]. In ethanol induced gastric damage in the form of gastric hemorrhage, oral administration of α-Bisabolol was realized to attenuate gastric damage and to provide cytoprotection in stomach. Prostaglandins and nitric oxide are known for their important role in protecting gastric mucosal and maintaining gastric blood flow.

To investigate their participation in α-Bisabolol action; rats were injected with indomethacin (cyclooxygenase inhibitor) and _L_-NAME (nitric oxide synthase inhibitor). The results revealed an abrogation in α-Bisabolol following their administration, indicating the involvement of prostaglandins and nitric oxide in the protection effect of α-Bisabolol. Furthermore, the decrease in α-Bisabolol action following the administration of K^+^_ATP_ channel blocker; glibenclamide offered evidence for the possible role of K^+^_ATP_ channel activation in mediating α-Bisabolol action [114]. However, a different study reported the involvement of another mechanism which is based on reverting the depletion of GSH caused by ethanol, where α-Bisabolol restored non-protein sulfydryl’s groups to which GSH belongs [115].

Furthermore, α-Bisabolol possesses antioxidant and anti-inflammatory properties which can protect the stomach. The antioxidant effect was demonstrated by causing an increase in SOD activity and a reduction in MDA levels. Whereas the anti-inflammatory effect was shown by the caused abolishment in MPO activity following ethanol injury leading to a decreased neutrophils influx into gastric mucosa [116]. Taken together, this demonstrates the anti-ulcerogenic activity of α-Bisabolol. The gastroprotective activity of α-Bisabolol is illustrated in Table 9.

## 9. α-Bisabolol and Nephroprotection

Ischemia-Reperfusion injury (I/R) can result in a catastrophic tissue dysfunction and cellular loss due to sudden restored blood flow to ischemic organ. Reperfusion injury is a multifaceted injury that extensively destruct injured organ by inducing exacerbated oxidative and inflammatory response [117].

In a model of ischemia performed on rats’ kidneys, α-Bisabolol was found to attenuate all induced biochemical alterations. For instance, α-Bisabolol abolished the increase in KIM-1 (kidney injury molecule-1), urinary osmolality, plasmatic creatinine, urea, uric acid, creatinine, proteinuria, and microalbuminuria. Additionally, α-Bisabolol protected against morphological changes including vacuolization, glomerular congestion and proteinaceous depositions in proximal tubules and external medulla [118].

The nephroprotection effect of α-Bisabolol was also tested in an in vitro model of I/R using HK2 cells. I/R caused significant cell death by both apoptosis and necrosis displayed by intense Annexin-V and (7AAD+/Anx+) staining. Cells treated with α-Bisabolol had a remarkable lower population of dead cells labelled with Annexin-V and (7AAD+/Anx+). α-Bisabolol reverted the increase in ROS and TBARS, enhanced GSH activity and most importantly induced NADPH production by inhibiting its oxidative degradation by NADPH oxidase, thus demonstrating an antioxidant defensive mechanism.

This was further validated by measuring NADPH oxidase gene (NOX4) expression, which was reduced after α-Bisabolol treatment [119]. The nephroprotective role of α-Bisabolol requires more extensive research to provide additional evidence on the effectiveness of α-Bisabolol and the mechanistic modulations against kidney injury. The nephroprotective effects of α-Bisabolol and the underlying mechanisms are summarized in Table 10.

## 10. Anti-Inflammatory Effects of α-Bisabolol

Inflammation is a physiological response that includes an array of cellular and molecular reactions designed for promoting cellular growth and tissue restoration after an injury. Chronic inflammation can have far reaching medical consequences and it is reported to be existed as an underlying mediator of various illnesses such as diabetes, cancer, and autoimmune diseases [120]. The anti-inflammatory effect of α-Bisabolol is presented in Table 11.

In an in vitro model resembling osteoarthritis, the anti-inflammatory effect of α-Bisabolol was assessed using human chondrocytes. The model was developed by exposing chondrocytes to advanced glycation end products (AGE) that are known to accumulate with aging in joint cartilage and activate the expressed receptor of AGE (RAGE). The treatment with α-Bisabolol suppressed the AGE-induced inflammatory response by reducing the expression of iNOS, COX-2, TNF-α, prostaglandin E2 (PGE2), nitrite, and IL-6. It also attenuated the extracellular matrix (ECM) catabolism observable by the upregulation of collagen II and aggrecan with a concomitant downregulation of MMP13 and ADAMT-S5 levels. In investigating the signaling pathways, α-Bisabolol inhibited the activation of nuclear factor kappa B (NF-κB) as well as the nuclear translocation of P65. Furthermore, it suppressed the AGE-induced mitogen-activated protein kinase (MAPK) signaling by decreasing the phosphorylation of c-Jun N-terminal kinase (JNK) and p38 [121].

To improve the anti-inflammatory actions of α-Bisabolol, a drug delivery system was developed using lipid-core nanocapsules (LNC). α-Bisabolol loaded nanoparticles (α-bis-LNC) showed a protective effect on the LPS induced leukocytes influx where it decreased the inflammatory cell migration and neutrophils accumulation in the bronchoalveolar lavage fluid confirmed by the quantified level of myeloperoxidase (MPO) which showed an activity lower than that observed in the LPS challenged mice.

Moreover, α-bis-LNC reversed the LPS induced increase in cytokine levels which was evident by the reduced macrophage inflammatory protein-2 (MIP-2) and chemokine (KC) levels. Animals treated with α-bis-LNC exhibited a decrease in the phosphorylated forms of ERK, p38 and JNK along with an abolished airway hyperreactivity (AHR) [122]. Similarly, α-Bisabolol caused a decrease in LPS-induced TNFα and IL-1β production in human myometrium samples [123]. Consistent findings were reported regarding the efficiency of α-Bisabolol in attenuating inflammatory response by decreasing the release of TNF-α following an inflammatory trigger [4].

In a systemic model of infection induced by cecal ligation and puncture (CLP), α-Bisabolol administration significantly reduced the number of recruited leukocytes in mice peritoneal cavity and the release of nitrite in the peritoneal exudate and protected against mortality associated with the systemic infection [124]. Moreover, treating LPS- stimulated macrophages with α-Bisabolol caused a decrease in NO, PGE2, iNOS and COX-2 expression. The anti-inflammatory effect of α-Bisabolol was envisaged to be mediated by the inhibition of NF-κB and activator protein-1 (AP-1) signaling cascade, and it was further elucidated by α-Bisabolol-induced suppression of ERK and p38 phosphorylation [125]. The topical application of α-Bisabolol demonstrated a remarkable inhibition of pro-inflammatory cytokines in 12-O-tetradecanoyl-phorbol-13-acetate (TPA) induced skin inflammation in mice [126].

## 11. The Antioxidant Actions of α-Bisabolol

Oxidative stress occurs when the production of free radicals exceeds the antioxidant defense resulting in an imbalance between the production and the stabilizing agents. The excessively formed reactive oxygen species interact with biomolecules including proteins, lipids and DNA causing deleterious changes and cellular degeneration [127]. Phytochemicals have a major role as free radical scavengers which can depress the level of produced reactive oxygen species and contribute in treating as well as preventing the development of many chronic diseases [128].

α-Bisabolol was shown to exert an antioxidant effect evaluated by biochemical tests including luminol-amplified chemiluminescence (LACL) on induced human polymorphonuclear neutrophils burst by *Candida albicans* and soluble stimulants (N-formyl-methionyl-leucyl-phenylalanine, fMLP). The LACL inhibition was apparent after α-Bisabolol treatment. Test on cell-free systems using H2O2/HOCl(-) and 3-morpholino-sydnonimine (SIN-1), the hydrolysis of which leads to the formation of superoxide anions as a result of oxygen reduction, exhibited an oxidation scavenging action of α-Bisabolol by reducing the burden of produces free radicals [129].

Additionally, 2,2-diphenyl-1-picryl-hydrazyl-hydrate (DPPH) test revealed that α-Bisabolol has an antioxidant activity with a reported IC50 value of >10,000 μg/mL. The ABTS radical scavenging test illustrated the antiradical potential of α-Bisabolol with a similar IC50 value to that reported in DPPH rest [130]. These finding are summarized in Table 12.

## 12. Toxicity Assessment of α-Bisabolol

Phytochemicals are generally classified as safe compounds as they are present in plants and produced naturally. However, the risk of toxicity assessment requires full consideration to eliminate toxic reactions and ensure not exceeding the recommended dose [131]. There is a good number of studies that assessed the toxicity of α-Bisabolol based on different in vivo models, yet the clinical efficacy and safety remains scarce and to be determined. The acute toxicity of α-Bisabolol was reported in mice and rats with oral LD50 of 15.1 mL/kg and 14.9 mL/kg, respectively, where symptoms such as sedation, ataxia, dyspnea, apathy, and ruffled fur were observed when exceeding aforementioned doses. The sub-chronic toxicity was assessed on rats based on 28 days duration of repeated dose and No Observed Adverse Effects level (NOAEL) of 200 mg/kg/day was determined. This indicates that α-Bisabolol has good oral compatibility in mice and rats with incompatibility reaction occurring only at high doses. The intraperitoneal LD50 was determined to be 0.633 g/kg in mice with symptoms of trembling, staggering gait and hepatic adhesions and α-Bisabolol deposits in sacrificed animals. However, inhalational exposure to aerosolized α-Bisabolol caused no deaths or lesions in necropsy.

The dermal irritation of α-Bisabolol was tested on rabbits by applying occlusive patches containing α-Bisabolol on their back and flank. Slight erythema was developed after the initial 4 h but was subsided with time. In human repeat insult patch test (HRIPT), there was no sensitization induced by α-Bisabolol with a documented No Expected Sensitization Induction Level (NESIL) of 5500 μg/cm^2^ [132]. Moreover, α-Bisabolol was found to be non-photoallergenic and no phototoxicity is reported post topical application of 5% and 0.5% α-Bisabolol solution on shaved guinea pigs exposed to UV radiation [133]. T

The mutagenicity of α-Bisabolol was evaluated in Salmonella/microsome assay which showed that α-Bisabolol did not increase the number of his^+^ revertant bacteria colonies when compared with the control values [134]. Furthermore, the developmental and reproductive toxicological effects of α-Bisabolol were investigated on pregnant rats and a (NOAEL) was determined to be 980 mg/kg/day with no prenatal development effects. Whereas the dose of 1000 mg/kg was realized to increase the occurrence of resorption rate and to reduce the fetal number, body weight and feed intake as well [132].

In addition, α-Bisabolol was shown to protect the fertility of mice against daunorubicin, an inducer of free radicals. All mice treated with α-Bisabolol had their fertilization capacity maintained with sperm motility and concentration not affected. This reflects the chemoprotective potential of α-Bisabolol [135].

## 13. Conclusions

It is irrefutable that plants can provide a vast reservoir for biologically active natural compounds which can be integrated in novel drugs discovery. There is increased evidence for the use of α-Bisabolol as a potential therapeutic agent and nutraceutical. This is evident in the spurred growth in the in vitro and in vivo studies evaluating the bioefficacy of α-Bisabolol, where numerous preclinical studies have provided a solid basis for its efficacy against human diseases.

As discussed in this review, α-Bisabolol has shown therapeutic potential in several pathological conditions and possesses a wide range of biological activities putatively propitious for clinical applications such as the anticancer, antimicrobial, and anti-inflammatory. It also demonstrated a particular protective effect against various disorders affects different organ systems including nervous and cardiovascular. Conceivably, the underlying mediating mechanism for the exerted bio-efficiency of α-Bisabolol is anticipated via the modulation of signaling pathways and involved molecules. This review summarized the studies based on which, α-Bisabolol might be one of the potent plants derived agent for application as nutraceutical or pharmaceutical with pharmacological rationale and molecular mechanisms.

## Figures and Tables

**Table 1:** Percentage occurrence of α-Bisabolol in different plants.

Plants	Percentage Occurrence	Reference
*Stachys lavandulifolia* Vahl. (*Lamiaceae*)	56.4	[4]
*Vanillosmopsis arborea* Barker (*Asteraceae*)	91.02	[5]
*Nectandra megapotamicav* (Spreng.) Mez. (*Lauraceae*)	93.7	[6]
*Nepeta graciliflora* Benth. (*Lamiaceae*)	8.97	[7]
*Lantana achyranthifolia* Desf. (*Verbenaceae*)	11.23	[8]
*Hymenocrater yazdianus, Stachys obtusicrena Boiss*, and *Nepeta asterotricha*	23.5%	[9]
*Tanacetum walteri* (*Anthemideae*-*Asteraceae*)	6.3	[10]
*Licaria, Nectrandra* and *Ocotea* Species (*Lauraceae*)	59.7–93.7	[11]
*Semenovia suffruticosa*	13.3	[12]
*Genus Matricaria*	29–81	[2]
*Algerian Eryngium tricuspidatum* L.	32.6	[13]
*Eupatorium adenophorum*	9.53	[14]
Candeia Tree (*Eremanthus erythropappus* (DC) McLeisch)	66–91	[15]
*Amomum biflorum*	16.0	[16]
*Acanthospermum hispidum* (*Asteraceae*)	11.4	[17]
*Helietta longifoliata*	7.24	[18]
*Betonica grandiflora* Willd.	4.9	[19]
*Rhaponticum acaule* DC	4.8	[20]
*Vismia macrophylla*	14.9	[21]
*Micromeria inodora* (Desf.) Benth.	2.9	[22]
*Ganoderma lucidum* and *Spongiporus leucomallellus*	2	[23]
*Laserpitium zernyi*	30.9	[24]
Araçá *(Psidium guineense* Sw.)	6.5–18.1	[25]
*Psidium myrtoides O. Berg* (*Myrtaceae*)	5.3	[26]
*Ferula hermonis* Boiss	11.1	[27]
*Plinia cerrocampanensis*	42.8	[28]
*Arnica longifolia*, *Aster hesperius*, and *Chrysothamnus nauseosus*	8.2	[29]
*Matricaria chamomilla* L.	56.86	[30]
*Teucrium polium* L.	24.6	[31]
*Angelica purpurascens* (Avé-Lall.) Gill	22.93	[32]
*Ferula asafoetida*	9.75	[33]

**Table 2:** * Physicochemical properties of α-Bisabolol.

PubChem CID	1549992
Molecular Formula	C15H26O
Synonyms	(+)-α-Bisabolol, D-α-Bisabolol, (2R)-6-Methyl-2-(4-methyl-3-cyclohexenyl)-5-heptene-2-ol, Dragosantol, Camilol, Hydagen B, (+)-6R,7R-α-Bisabolol,
Molecular Weight	222.37
XLogP3-AA	3.8
Hydrogen Bond Donor Count	1
Hydrogen Bond Acceptor Count	Rotatable Bond Count
Exact Mass	222.198365449
Monoisotopic Mass	222.198365449
Topological Polar Surface Area	20.2 Å^2^
Heavy Atom Count	16
Formal Charge	0
Complexity	284
Isotope Atom Count	0
Defined Atom Stereocenter Count	2
Undefined Atom Stereocenter Count	0
Defined Bond Stereocenter Count	0
Undefined Bond Stereocenter Count	0
Covalently-Bonded Unit Count	1
Solubility	1.688 mg/L @ 25 °C (est), Practically insoluble or insoluble in water, slightly soluble in ethanol
Density	0.922–0.931
LogP	5.070 (est)
Refractive Index	1.491–1.500
Food additive class	Flavoring agent

* Compiled from the PubChem substances.

**Table 3:** Therapeutic effects and demonstrated mechanisms of α-Bisabolol in skin disorders.

Skin Disorders				
Compound	Dose/Route/ Duration	Model	Major Mechanisms	Reference
α-Bisabolol	1% topical spray	Randomized controlled trial on chronic venous leg ulcer patients	↓ wound and ulcer surface area ↑ healing speed	[37]
α-Bisabolol	0.3 g/100 g cream twice daily for 8 weeks	Prospective, randomized, reference-controlled, double-blind, two-center and four-armed parallel group study on patients with atopic dermatitis	↓ pruritis, inflammation ↑ healing	[39]
α-Bisabolol	1% cream once-daily for 30 days	Single-center, single-arm, prospective, open-label study on patients with melasma	↓ melasma area and severity index (MASI) score ↑ patient satisfaction	[40]

Symbols indications: ↑; increase, ↓; decrease.

**Table 4:** Neuroprotective effects and underlying mechanisms of α-Bisabolol.

Neuroprotective				
Compound	Dose/Route/ Duration	Model	Major Mechanisms	Reference
α-Bisabolol	5, 25, and 250 μmol/L for 7 days	Rotenone (500 μmol/L) induced neurotoxicity in *Drosophila*	↓ mortality and motor deficits, ↓ thiol level ↑ SOD, CAT and Keap1	[46]
α-Bisabolol	50 mg/kg i.p, 30 min before rotenone for 4 weeks	Rotenone (2.5 mg/kg) induced Parkinson’s disease	⇥ neuronal loss, ↓ MDA, ↑ GSH, SOD and CAT, ↓ glial activation, ↓ IL-1β, IL-6, TNF-α, iNOS and COX-2, ↑ Bcl-2, ↓ Bax, caspases-3, 9 and cytochrome-C, restored ATP and MC-I activity	[47]
α-Bisabolol	5, 10 μg/mL for 2 h in N2a cells and 25, 50 and 100 μg/mL in elegans	Aβ_25–35_ peptide (50 μM for 24 h) induced toxicity in N2a cells and *Caenorhabditis elegans* CL4176 and CL2006	⇥ cholinesterase and β-secretase, ↓ ROS and RNS ↓ Bax and caspase-3 ↓ ace-1, hsp-4 and Aβ genes	[49]
α-Bisabolol	5 mg/mL for 2 h	Aβ_25–35_ peptide (50 μM for 24 h) induced toxicity in PC12 cells	↓ Aβ aggregation ↑ cell survival	[50]
α-Bisabolol	5 and 10 μg/mL for 2 h	Aβ_25–35_ peptide (50 μM for 24 h) induced toxicity in Neuro-2a cells	↓ ROS and RNS, ↓ β-secretase and AchE activities, ↓ Bax, caspase3, and ↑ Bcl-2	[51]
α-Bisabolol β-D-fucopyranoside	10–50 μg/mL	Aβ_25–35_ (100 μM for 24 h, 48 h, 96 h, 9 d) induced toxicity in Neuro 2a cells	Inhibited AChE, ↓ H_2_O_2_ and OH^•^, ↓ Aβ aggregation ↑ cell survival	[52]
α-Bisabolol	50, 100 and 200 mg/kg/day, p.o	Permanent occlusion of the middle cerebral artery induced cerebral ischemia in mice	↓ infarct size, ↑ motor performance, ↑ crossings and rearings	[54]

Symbols indications: ↑; increase, ↓; decrease, ⇥; activity inhibition.

**Table 5:** Anticancer effects and demonstrated mechanisms of α-Bisabolol in the experimental models.

Anticancer Effects				
Compound	Dose/Route/Duration	Model	Major Mechanisms	Reference
α-Bisabolol	0 to 32 μmol/L for 24 h	EC cell lines including RL95-2, ECC001 and ECC003 cells	(-) EC cells growth ↑ caspase-3, ↑ PARP ↓ XIAP, COX-2 ↑ radiotherapy effect	[59]
α-Bisabolol	35, 45 or 55 μM for C6 glioma cells and 55, 65 or 75 μM for U138-MG	U138-MG human and C6 rat glioma cell lines	↓ cell viability ↑ ecto5′-NT/CD73	[61]
α-Bisabolol	100 and 250 μM	Human and rat glioma cell lines	↓ cell viability ↑ Cytochrome-C	[62]
α-Bisabolol	1 mM	Human prostate cancer cell line PC-3, human cervical carcinoma cell line Hela, human esophageal ECA-109, and human liver carcinoma cell line HepG2	↑ caspases 3, 8 and 9 ↑ cytochrome-C ↑ Bax, Bid, ↓ Bak and Bcl-2, p53, ↑ NF-κB and Fas	[64]
α-Bisabolol	0 t0 100 μM for 24 h (IC50 = 15 µM)	NSCLC cell line A549	(-) migration of A549 cells, (-) PI3K/AKT, ↑ apoptotic cells ↑ Bax, ↓ Bcl-2, triggers G2/M cell cycle arrest	[66]
α-Bisabolol	0–250 μM 1000 mg/kg, (21–27 mg/mouse) once a week for 3 weeks	KLM1, Panc1, MIA Paca2 and KP4 human pancreatic cancer cell lines, BALB ⁄ c nude mice xenograft model inoculated with KLM1 and KP4 cells (1 × 10^7^ s.c.) in femoral area	↓ cells viability, ↑ apoptosis, (-) AKT, ↑ EGR1, ↓ tumor volume and weight	[69]
α-Bisabolol	1.56 μM	KLM1, KP4 and Panc1 human pancreatic cancer cell lines	(-) motility of cells ↑ KISS1R, MTSS1 and TIMP2	[70]
α-Bisabolol	0, 3, 15, 30, 60, 125, 250 μM	CML-T1primary human acute leukemia cell line	↓ cells viability, induced apoptosis	[72]
α-Bisabolol	300 µL intra-mammary injection (3.6 mg and 10 mg per mouse)	HER2/neu transgenic mice	↓ tumor mass ↓ HER2/neu, Fgf and Birc5 ↑ natural killer cytotoxicity	[73]
α-Bisabolol and its derivative	62.5 μM and 125 μM, 1000 mg/kg	KLM1 and Panc1 human pancreatic cancer cell lines, BALB/c nude mice implanted with KLM1; cells (1 × 10^7^ cells/100 μL, s.c.) into femoral area	↑ cell death ↓ volume of tumor ↓ CEA and CA19-9, ↓ disseminated tumorous nodules ⇥ AKT	[74]
α-Bisabolol β-D-fucopyranoside	IC50 > 100 μM	human lung carcinoma (A549), colon adeno-carcinoma (DLD-1), breast adeno-carcinoma (MCF-7), melanoma (SK-MEL-2), ovary teratocarcinoma (PA-1), prostate adeno-carcinoma (PC-3), pancreas adeno-carcinoma (PANC 05.04), glioma (U-251), glioblastoma (U-87) and murine glioma (GL-261)	↑ α-Bisabolol cytotoxicity ↑ BBB penetration ↑ α-Bisabolol lipophilicity	[75]
α-Bisabolol-based thiosemicarbazones compounds	0.25 to 250 mg/mL	Melanoma UACC-62, breast MCF-7, breast resistant NCI-ADR, lung NCI-460, leukemia K-562, ovarian OVCAR, prostate PCO-3, and colon HT29 cell lines	(-) cell growth	[76]

Symbols indications: ↑; increase, ↓; decrease, (-); reduce, ⇥; activity inhibition.

**Table 6:** The anti-nociceptive actions of α-Bisabolol.

Antinociceptive Effects				
Compound	Dose/Route/ Duration	Model	Major Mechanisms	Reference
α-Bisabolol	200 mg/kg p.o	IONX-induced acute orofacial neuropathic pain in rats	↓ mechanical hypersensitivity ↑ pain threshold	[78]
α-Bisabolol	50 mg/kg, p.o	FCA (25 μL, i.p.) and PLSN induced pain in mice	↓ mechanical and thermal hyperalgesia ↓ gliosis, ↑ IL-10, ↓ TNF-α	[79]
α-Bisabolol	25 or 50 mg/kg, p.o 1 h before the local injection of inducing agents	Formalin (20 μL of 2% s.c.), capsaicin (20 μL of 2.5 µg, s.c.) or glutamate (40 μL of 25 mM, s.c.) induced orofacial nociception Carrageenan (100 µL of 1%*w*/*v* intrapleural) induced pleurisy in mice	↓ orofacial pain ↓ TNF-α	[4]
α-Bisabolol	30, 56, 100, and 180 mg/kg p.o. of α-Bisabolol alone α-Bisabolol -diclofenac (5.1, 10.3, 20.6, and 41.2 mg/kg)	Formalin (50 µL of 1%, s.c.) induced nociception Carrageenan (100 µL of a 1%, s.c.)-induced inflammation in rats	↓ nociception ↓ paw volume ↓ hemorrhagic erosion	[86]
α-Bisabolol	25, 50, 100 and 200 mg/kg p.o	Carrageenan (20 μL 1%*w*/*v*, intraplantar injection), dextran (20 μL of 0.15%, *w*/*v*), histamine (200 μg/paw) or serotonin (200 μg/paw) induced inflammation, formalin (20 μL of 1%) induced nociception, acetic acid (0.1 mL/10 g of 0.6% solution)-induced abdominal writhing in rats	↓ paw licking ↓ edema volume ↓ abdominal writhing ↓ leukocytes migration ↓ MPO release ↓ TNF-α	[83]
α-Bisabolol	100, 200, or 400 mg/kg p.o., or 50, 100, or 200 mg/mL topical 60 min before injection	Formalin (20 μL of 1.5% s.c.), cinnamaldehyde (13.2 μg/lip) induced nociception in rodents	↓ face rubbing ↓ head flinching	[80]
α-Bisabolol	50, 100 or 200 mg/kg, p.o	Cyclophosphamide (400 mg/kg, i.p.), mustard oil (50 μL/animal intracolonic) induced visceral nociception in mice	↓ visceral pain	[84]
α-Bisabolol	50–200 mg/mL ointment	Hypertonic saline (20 μL of 5 M NaCl)-induced corneal nociception in mice	↓ eye wiping	[85]
α-Bisabolol nanocapsules	100 or 200 mg/mL	Hypertonic saline (20 μL of 5M NaCl)-induced corneal nociception in mice	↓ eye wiping	[87]
α-Bisabolol	50, 100 or 200 mg/kg, p.o	Acetic acid (0.6%, i.p.), Capsaicin (50 μL/animal, intracolonic), Formalin (10%, 10 μL/animal, intracolonic), (0.75%, 50 μL/animal, intracolonic) induced visceral nociception in mice	↓ abdominal constrictions ↓ pain-related behavior	[81]
α-Bisabolol	0.5, 1, 5 and 10 mM	Supramaximal stimulation consisted of 50–100 μs isolated rectangular voltage pulses applied on mice sciatic nerves	↓ nerve excitability	[82]
α-Bisabolol	1–0.5% mouthwash	postoperative complications of maxillofacial surgeries, a randomized, controlled, triple-blind clinical trial	↓ pain during brushing ↓ lesion wiping	[88]

Symbols indications: ↑; increase, ↓; decrease.

**Table 7:** Cardioprotective effects and mechanisms of α-Bisabolol.

Cardioprotective Effects				
Compound	Dose/Route/ Duration	Model	Major Mechanisms	Reference
α-Bisabolol	25 mg/kg, i.p for 10 days	Isoproterenol (85 mg/kg, s.c. for 2 days) induced myocardial infarction in rats	↓ LDH, ↓ infarct size ↓ TBARS, ↑ SOD, CAT, ↓ β-glucuronidase, β-galactosidase, cathepsin-B &D, ↓ TNF-α, IL-6, IL-1β, iNOS and COX-2, ↑ IL-10, ↓ NLRP3, (-) NFκB/MAPK, ↑ Beclin-1, LC3BI/II, ↓ p-mTOR	[91]
α-Bisabolol	25 mg/kg, i.p daily for 10 days	Isoproterenol (85 mg/kg, s.c. for 2 days) induced myocardial infarction in rats	↑ CK and LDH, ↓ LOOH, TBARS, ↑ SOD, catalase and GSH, ↓ Ca^2+^ overload ↓ mitochondrial swelling, ↑ ATP, ↑ ICDH, SDH, MDH, α-KGDH, and complexes I-IV, ↓ Bax, P53, APAF-1, active caspase-3 and 9, ↑ Bcl-2	[92]
α-Bisabolol	25 mg/kg, i.p daily for 10 days	Isoproterenol (85 mg/kg, s.c. for 2 days) induced myocardial infarction in rats	↓ CK ↓ TBARS and LOOH ↑ GSH and vitamin-C ↓ HR, SBP and DBP	[93]

Symbols indications: ↑; increase, ↓; decrease, (-); reduce.

**Table 9:** Effects of α-Bisabolol in preclinical models of gastrointestinal diseases.

Dose/Route/Duration	Model	Major Mechanisms	Reference
100 mg/kg p.o.	Ethanol (96%, 1 mL per animal) induced gastric damage in rats	↓ gastric damage	[114]
100 or 200 mg/kg p.o.	Ethanol (0.2 mL/animal p.o.) and Indomethacin (20 mg/kg p.o.) induced ulcer model in mice	↓ gastric lesions ↑ GSH	[115]
100 and 200 mg/kg, p.o.	Ethanol (0.2 mL) induced gastric lesion in mice	↓ MDA, MPO, ↑ SOD, ↓ neutrophils influx	[116]

Symbols indications: ↑; increase, ↓; decrease.

**Table 10:** Nephroprotective effects of α-Bisabolol.

100 mg/kg p.o. and 500, 250, 125, 62.5 and 31.25 μM	Clamping of the renal artery in the left kidney for 60 min. in rats and Ischemia/reperfusion model on tubular epithelial cells (LLC-MK_2_) by anaerobic chamber method	↓ creatinine, urea, uric acid ↓ urinary osmolality ↓ FeNa^+^, FeK^+^, FeCl^−^ ↓ microalbuminuria ↓ KIM-1, ↓ TBARS, ↑ GSH ↑ cell viability	[118]
1000, 500, 250, 125, 62.5 and 31.25 μM	Ischemia/reperfusion model on human tubular kidney cells (HK2) by anaerobic chamber method	↑ cell viability, ↓ apoptosis, ↓ TBARS, (-) NADPH oxidase ↑ GSH, ↓ NOX4, ↑ ΔΨm, ↓ KIM-1	[119]

Symbols indications: ↑; increase, ↓; decrease, (-); reduce.

**Table 11:** Anti-inflammatory effects of α-Bisabolol in different experimental models.

Dose/Route/ Duration	Model	Major Mechanisms	Reference
Cells treated with: 2.5, 5, 10 μM for 24 h Mice treated with 30 mg/kg/day p.o daily for 8 weeks	AGEs (50μg/mL for 2 h) induced OA in chondrocytes and Destabilization of the medial meniscus in mice	↓ iNOS, COX-2, TNF-α, p65 PGE2, nitrite, IL-6, ↓ MMP13 ↑ collagen II, aggrecan ADAMT-S5, ↓ NF-κB, IκBα, ↓ pJNK, ↓ p-p38, ↑ chondrocytes and proteoglycans	[121]
30, 50, or 100 mg/kg, p.o, 4 h before LPS	LPS (25 µg/25 µL intranasal) induced acute lung inflammation in mice	↓ neutrophils, ↓ MPO, ↓ AHR, ↓ elastance, ↓ MIP-2 and KC ↓ alveolar wall thickening, inflammatory cell infiltration, alveolar hemorrhage, and lung tissue damage	[122]
560, 860 and 1200 μM	LPS (10 μg/mL for 24 h) induced inflammation in human myometria biopsies	↓ TNF-α, IL-1β	[123]
50 mg/kg, p.o	Carrageenan (100 μL of 1% (*w*/*v*)) induced pleurisy in mice	↓ TNF-α	[4]
In vitro: 0.5, 1, 3, 10, 30, or 90 μg/mL and in vivo: 50, 100, or 200 mg/kg p.o 1 h before surgery	Zymosan (1 mg/cavity, i.p.) induced neutrophils in peritoneal cavity of mice and Cecal ligation and puncture induced systemic infection	↑ phagocytosis of neutrophils, ↓ leukocytes, ↓ NO, ↓ mortality ↓ colony forming units	[124]
25 and 100 μM for 2h	LPS (500 ng/mL) induced inflammatory response in RAW264.7 macrophages cells	↓ NO, PGE2, ↓ iNOS, COX-2, ↓ NF-jB, AP-1, ↓ pERK, p-p38	[125]

Symbols indications: ↑; increase, ↓; decrease.

**Table 12:** The antioxidant actions of α-Bisabolol.

Dose/Route/ Duration	Model	Major Mechanisms	Reference
1.9 to 31 g/m	*Candida albicans* and *fMLP* induced Human polymorphonuclear neutrophils respiratory, Bursts and ROS production	↑ LACL inhibition	[129]
1000 μg/mL to 62.5 μg/mL	In vitro tests (DPPH and ABTS)	↓ concentration of free radicals	[130]

Symbols indications: ↑; increase, ↓; decrease.

## Data Availability

This is a review article, and majority of the articles referred herein this manuscript has been suitably cited in the manuscript.

## References

[1] Da Silveira e Sá Rde C., Andrade L.N., de Sousa D.P. (2015). Sesquiterpenes from essential oils and anti-inflammatory activity. Nat. Prod. Commun..

[2] Sharifi-Rad M., Nazaruk J., Polito L., Morais-Braga M.F.B., Rocha J.E., Coutinho H.D.M., Salehi B., Tabanelli G., Montanari C., Del Mar Contreras M. (2018). Matricaria genus as a source of antimicrobial agents: From farm to pharmacy and food applications. Microbiol. Res..

[3] McKay D.L., Blumberg J.B. (2006). A review of the bioactivity and potential health benefits of chamomile tea (*Matricaria recutita* L.). Phytother. Res..

[4] Barreto R.S.S., Quintans J.S.S., Amarante R.K.L., Nascimento T.S., Amarante R.S., Barreto A.S., Pereira E.W.M., Duarte M.C., Coutinho H.D.M., Menezes I.R.A. (2016). Evidence for the involvement of TNF-α and IL-1β in the antinociceptive and anti-inflammatory activity of *Stachys lavandulifolia* Vahl. (Lamiaceae) essential oil and (-)-α-Bisabolol, its main compound, in mice. J. Ethnopharmacol..

[5] Rodrigues F.F.G., Colares A.V., Nonato C.F.A., Galvão-Rodrigues F.F., Mota M.L., Moraes Braga M.F.B., Costa J. (2018). In vitro antimicrobial activity of the essential oil from Vanillosmopsis arborea Barker (Asteraceae) and its major constituent, α-Bisabolol. Microb. Pathog..

[6] Farias K.S., Kato N.N., Boaretto A.G., Weber J.I., Brust F.R., Alves F.M., Tasca T., Macedo A.J., Silva D.B., Carollo C.A. (2019). Nectandra as a renewable source for (+)-α-Bisabolol, an antibiofilm and anti-Trichomonas vaginalis compound. Fitoterapia.

[7] Sharma P., Shah G.C., Sharma R., Dhyani P. (2016). Chemical composition and antibacterial activity of essential oil of *Nepeta graciliflora* Benth. (Lamiaceae). Nat. Prod. Res..

[8] Hernández T., Canales M., Avila J.G., García A.M., Martínez A., Caballero J., de Vivar A.R., Lira R. (2005). Composition and antibacterial activity of essential oil of *Lantana achyranthifolia* Desf. (Verbenaceae). J. Ethnopharmacol..

[9] Masoudi S., Rustaiyan A., Mohebat R., Mosslemin M.H. (2012). Composition of the essential oils and antibacterial activities of *Hymenocrater yazdianus*, *Stachys obtusicrena* and *Nepeta asterotricha* three Labiatae herbs growing wild in Iran. Nat. Prod. Commun..

[10] Ghaderi A., Sonboli A. (2019). Chemical composition and antimicrobial activity of the essential oil of Tanacetum walteri (Anthemideae-Asteraceae) from Iran. Nat. Prod. Res..

[11] Xavier J., Alves N.S.F., Setzer W.N., da Silva J.K.R. (2020). Chemical diversity and biological activities of essential oils from Licaria, Nectrandra and Ocotea Species (Lauraceae) with occurrence in Brazilian biomes. Biomolecules.

[12] Soltanian S., Mohamadi N., Rajaei P., Khodami M., Mohammadi M. (2019). Phytochemical composition, and cytotoxic, antioxidant, and antibacterial activity of the essential oil and methanol extract of *Semenovia suffruticosa*. Avicenna J. Phytomed..

[13] Merghache D., Boucherit-Otmani Z., Merghache S., Chikhi I., Selles C., Boucherit K. (2014). Chemical composition, antibacterial, antifungal and antioxidant activities of Algerian *Eryngium tricuspidatum* L. essential oil. Nat. Prod. Res..

[14] Kurade N.P., Jaitak V., Kaul V.K., Sharma O.P. (2010). Chemical composition and antibacterial activity of essential oils of *Lantana camara*, *Ageratum houstonianum* and *Eupatorium adenophorum*. Pharm. Biol..

[15] Alves Gomes Albertti L., Delatte T.L., Souza de Farias K., Galdi Boaretto A., Verstappen F., van Houwelingen A., Cankar K., Carollo C.A., Bouwmeester H.J., Beekwilder J. (2018). Identification of the Bisabolol synthase in the endangered candeia tree (*Eremanthus erythropappus* (DC) McLeisch). Front Plant Sci..

[16] Singtothong C., Gagnon M.J., Legault J. (2013). Chemical composition and biological activity of the essential oil of *Amomum biflorum*. Nat. Prod. Commun..

[17] Alva M., Popich S., Borkosky S., Cartagena E., Bardón A. (2012). Bioactivity of the essential oil of an argentine collection of *Acanthospermum hispidum* (Asteraceae). Nat. Prod. Commun..

[18] De Moura N.F., Simionatto E., Porto C., Hoelzel S.C., Dessoy E.C., Zanatta N., Morel A.F. (2002). Quinoline alkaloids, coumarins and volatile constituents of *Helietta longifoliata*. Planta Med..

[19] Yousefi M., Gandomkar S., Habibi Z. (2012). Essential oil from aerial parts of of *Betonica grandiflora* Willd. from Iran. Nat. Prod. Res..

[20] Boussaada O., Ammar S., Saidana D., Chriaa J., Chraif I., Daami M., Helal A.N., Mighri Z. (2008). Chemical composition and antimicrobial activity of volatile components from capitula and aerial parts of Rhaponticum acaule DC growing wild in Tunisia. Microbiol. Res..

[21] Buitrago A., Rojas J., Rojas L., Velasco J., Morales A., Peñaloza Y., Díaz C. (2015). Essential oil composition and antimicrobial activity of *Vismia macrophylla* leaves and fruits collected in Táchira-Venezuela. Nat. Prod. Commun..

[22] Benomari F.Z., Djabou N., Medbouhi A., Khadir A., Bendahou M., Selles C., Desjobert J.M., Costa J., Muselli A. (2016). Chemical variability and biological activities of essential oils of micromeria inodora (Desf.) Benth. from Algeria. Chem. Biodivers..

[23] Campos Ziegenbein F., Hanssen H.P., König W.A. (2006). Secondary metabolites from *Ganoderma lucidum* and *Spongiporus leucomallellus*. Phytochemistry.

[24] Popović V., Petrović S., Pavlović M., Milenković M., Couladis M., Tzakou O., Duraki S., Niketić M. (2010). Essential oil from the underground parts of *Laserpitium zernyi*: Potential source of alpha-Bisabolol and its antimicrobial activity. Nat. Prod. Commun..

[25] Figueiredo P.L.B., Silva R.C., da Silva J.K.R., Suemitsu C., Mourão R.H.V., Maia J.G.S. (2018). Chemical variability in the essential oil of leaves of Araçá (*Psidium guineense* Sw.), with occurrence in the Amazon. Chem. Cent. J..

[26] Dias A.L.B., Batista H.R.F., Estevam E.B.B., Alves C.C.F., Forim M.R., Nicolella H.D., Furtado R.A., Tavares D.C., Silva T.S., Martins C.H.G. (2019). Chemical composition and in vitro antibacterial and antiproliferative activities of the essential oil from the leaves of Psidium myrtoides O. Berg (Myrtaceae). Nat. Prod. Res..

[27] Al-Ja’fari A.H., Vila R., Freixa B., Tomi F., Casanova J., Costa J., Cañigueral S. (2011). Composition and antifungal activity of the essential oil from the rhizome and roots of Ferula hermonis. Phytochemistry.

[28] Vila R., Santana A.I., Pérez-Rosés R., Valderrama A., Castelli M.V., Mendonca S., Zacchino S., Gupta M.P., Cañigueral S. (2010). Composition and biological activity of the essential oil from leaves of *Plinia cerrocampanensis*, a new source of alpha-Bisabolol. Bioresour. Technol..

[29] Tabanca N., Demirci B., Crockett S.L., Başer K.H., Wedge D.E. (2007). Chemical composition and antifungal activity of *Arnica longifolia*, *Aster hesperius*, and *Chrysothamnus nauseosus* essential oils. J. Agric. Food Chem..

[30] Tolouee M., Alinezhad S., Saberi R., Eslamifar A., Zad S.J., Jaimand K., Taeb J., Rezaee M.B., Kawachi M., Shams-Ghahfarokhi M. (2010). Effect of *Matricaria chamomilla* L. flower essential oil on the growth and ultrastructure of *Aspergillus niger* van Tieghem. Int. J. Food Microbiol..

[31] Sayyad R., Farahmandfar R. (2017). Influence of *Teucrium polium* L. essential oil on the oxidative stability of canola oil during storage. J. Food Sci. Technol..

[32] Alkan Türkuçar S., Aktaş Karaçelik A., Karaköse M. (2021). Phenolic compounds, essential oil composition, and antioxidant activity of *Angelica purpurascens* (Avé-Lall.) Gill. Turk. J. Chem..

[33] Niazmand R., Razavizadeh B.M. (2021). Ferula asafoetida: Chemical composition, thermal behavior, antioxidant and antimicrobial activities of leaf and gum hydroalcoholic extracts. J. Food Sci. Technol..

[34] Kamatou G.P.P., Viljoen A.M. (2010). A review of the application and pharmacological properties of α-bisabolol and α-bisabolol -rich oils. J. Am. Oil Chem. Soc..

[35] Hay R.J., Johns N.E., Williams H.C., Bolliger I.W., Dellavalle R.P., Margolis D.J., Marks R., Naldi L., Weinstock M.A., Wulf S.K. (2014). The global burden of skin disease in 2010: An analysis of the prevalence and impact of skin conditions. J. Invest. Derm..

[36] Mintie C.A., Singh C.K., Ahmad N. (2020). Whole fruit phytochemicals combating skin damage and carcinogenesis. Transl. Oncol..

[37] Solovăstru L.G., Stîncanu A., De Ascentii A., Capparé G., Mattana P., Vâţă D. (2015). Randomized, controlled study of innovative spray formulation containing ozonated oil and α-Bisabolol in the topical treatment of chronic venous leg ulcers. Adv. Ski. Wound Care.

[38] Licari A., Ruffinazzi G., M D.E.F., Castagnoli R., Marseglia A., Agostinis F., Puviani M., Milani M., Marseglia G.L. (2017). A starch, glycyrretinic, zinc oxide and Bisabolol based cream in the treatment of chronic mild-to-moderate atopic dermatitis in children: A three-center, assessor blinded trial. Minerva Pediatr..

[39] Arenberger P., Arenbergerová M., Drozenová H., Hladíková M., Holcová S. (2011). Effect of topical heparin and levomenol on atopic dermatitis: A randomized four-arm, placebo-controlled, double-blind clinical study. J. Eur. Acad. Derm. Venereol..

[40] Crocco E.I., Veasey J.V., Boin M.F., Lellis R.F., Alves R.O. (2015). A novel cream formulation containing nicotinamide 4%, arbutin 3%, Bisabolol 1%, and retinaldehyde 0.05% for treatment of epidermal melasma. Cutis.

[41] Nemelka O., Bleidel D., Fabrizi G., Camplone G., Occella C., Marzatico F., Pecis L., Bocchietto E. (2002). Experimental survey of a new topical anti-oxidant based on furfuryl palmitate in the treatment of child’s and baby’s dermatitis with eczema: Results from a multicenter clinical investigation. Minerva Pediatr..

[42] Han G., Ceilley R. (2017). Chronic wound healing: A review of current management and treatments. Adv. Ther..

[43] Villegas L.F., Marçalo A., Martin J., Fernández I.D., Maldonado H., Vaisberg A.J., Hammond G.B. (2001). (+)-epi-Alpha-Bisabolol [correction of bisbolol] is the wound-healing principle of *Peperomia galioides*: Investigation of the in vivo wound-healing activity of related terpenoids. J. Nat. Prod..

[44] Dugger B.N., Dickson D.W. (2017). Pathology of neurodegenerative diseases. Cold Spring Harb. Perspect. Biol..

[45] Sarkar S., Raymick J., Imam S. (2016). Neuroprotective and therapeutic strategies against Parkinson’s Disease: Recent perspectives. Int. J. Mol. Sci..

[46] Leite G.O., Ecker A., Seeger R.L., Krum B.N., Lugokenski T.H., Fachinetto R., Sudati J.H., Barbosa N.V., Wagner C. (2018). Protective effect of (-)-α-Bisabolol on rotenone-induced toxicity in *Drosophila melanogaster*. Can. J. Physiol. Pharm..

[47] Javed H., Meeran M.F.N., Azimullah S., Bader Eddin L., Dwivedi V.D., Jha N.K., Ojha S. (2020). α-Bisabolol, a dietary bioactive phytochemical attenuates dopaminergic neurodegeneration through modulation of oxidative stress, neuroinflammation and apoptosis in rotenone-induced rat model of Parkinson’s disease. Biomolecules.

[48] Murphy M.P., LeVine H. (2010). Alzheimer’s disease and the amyloid-beta peptide. J. Alzheimer’s Dis..

[49] Shanmuganathan B., Sathya S., Balasubramaniam B., Balamurugan K., Devi K.P. (2019). Amyloid-β induced neuropathological actions are suppressed by *Padina gymnospora* (Phaeophyceae) and its active constituent α-Bisabolol in Neuro2a cells and transgenic *Caenorhabditis elegans* Alzheimer’s model. Nitric. Oxide.

[50] Shanmuganathan B., Suryanarayanan V., Sathya S., Narenkumar M., Singh S.K., Ruckmani K., Pandima Devi K. (2018). Anti-amyloidogenic and anti-apoptotic effect of α-Bisabolol against Aβ induced neurotoxicity in PC12 cells. Eur. J. Med. Chem..

[51] Sathya S., Shanmuganathan B., Devi K.P. (2020). Deciphering the anti-apoptotic potential of α-Bisabolol loaded solid lipid nanoparticles against Aβ induced neurotoxicity in Neuro-2a cells. Colloids Surf. B Biointerfaces.

[52] Jeyakumar M., Sathya S., Gandhi S., Tharra P., Suryanarayanan V., Singh S.K., Baire B., Pandima Devi K. (2019). α-Bisabolol β-D-fucopyranoside as a potential modulator of β-amyloid peptide induced neurotoxicity: An in vitro &in silico study. Bioorg. Chem..

[53] Nour M., Scalzo F., Liebeskind D.S. (2012). Ischemia-reperfusion injury in stroke. Interv. Neurol..

[54] Fernandes M.Y.D., Carmo M., Fonteles A.A., Neves J.C.S., Silva A., Pereira J.F., Ferreira E.O., Lima N.M.R., Neves K.R.T., Andrade G.M. (2019). (-)-α-Bisabolol prevents neuronal damage and memory deficits through reduction of proinflammatory markers induced by permanent focal cerebral ischemia in mice. Eur. J. Pharm..

[55] Ok C., Woda B., Kurian E. (2018). The Pathology of Cancer.

[56] Pfeffer C.M., Singh A.T.K. (2018). Apoptosis: A target for anticancer therapy. Int. J. Mol. Sci..

[57] Darra E., Abdel-Azeim S., Manara A., Shoji K., Maréchal J.-D., Mariotto S., Cavalieri E., Perbellini L., Pizza C., Perahia D. (2008). Insight into the apoptosis-inducing action of α-Bisabolol towards malignant tumor cells: Involvement of lipid rafts and Bid. Arch. Biochem. Biophys..

[58] Passarello K., Kurian S., Villanueva V. (2019). Endometrial cancer: An overview of pathophysiology, management, and care. Semin. Oncol. Nurs..

[59] Fang D., Wang H., Li M., Wei W. (2019). α-Bisabolol enhances radiotherapy-induced apoptosis in endometrial cancer cells by reducing the effect of XIAP on inhibiting caspase-3. Biosci. Rep..

[60] Omuro A., DeAngelis L.M. (2013). Glioblastoma and other malignant gliomas: A clinical review. JAMA.

[61] Mendes F.B., Bergamin L.S., Dos Santos Stuepp C., Braganhol E., Terroso T., Pohlmann A.R., Guterres S.S., Battastini A.M. (2017). Alpha-bisabolol promotes glioma cell death by modulating the adenosinergic system. Anticancer Res..

[62] Cavalieri E., Mariotto S., Fabrizi C., de Prati A.C., Gottardo R., Leone S., Berra L.V., Lauro G.M., Ciampa A.R., Suzuki H. (2004). alpha-Bisabolol, a nontoxic natural compound, strongly induces apoptosis in glioma cells. Biochem. Biophys. Res. Commun..

[63] Ibrahim N., Aboulthana W., Sahu D.R. (2018). Hepatocellular carcinoma: Causes and prevention. UK J. Pharm. Biosci..

[64] Chen W., Hou J., Yin Y., Jang J., Zheng Z., Fan H., Zou G. (2010). alpha-Bisabolol induces dose- and time-dependent apoptosis in HepG2 cells via a Fas- and mitochondrial-related pathway, involves p53 and NFkappaB. Biochem. Pharm..

[65] Akbulut H., İncedayı S., Atasoy Ö. (2020). Non-small cell lung cancer and its treatment. Demiroglu Sci. Univ. Florence Nightingale Transplant. J..

[66] Wu S., Peng L., Sang H., Ping Li Q., Cheng S. (2018). Anticancer effects of α-Bisabolol in human non-small cell lung carcinoma cells are mediated via apoptosis induction, cell cycle arrest, inhibition of cell migration and invasion and upregulation of P13K/AKT signalling pathway. J. Buon.

[67] Kleeff J., Korc M., Apte M., Vecchia C., Johnson C., Biankin A., Neale R., Tempero M., Tuveson D., Hruban R. (2016). Pancreatic cancer. Nat. Rev. Dis. Primers.

[68] Yu J., Zhang S.S., Saito K., Williams S., Arimura Y., Ma Y., Ke Y., Baron V., Mercola D., Feng G.-S. (2009). PTEN regulation by Akt-EGR1-ARF-PTEN axis. EMBO J..

[69] Seki T., Kokuryo T., Yokoyama Y., Suzuki H., Itatsu K., Nakagawa A., Mizutani T., Miyake T., Uno M., Yamauchi K. (2011). Antitumor effects of α-Bisabolol against pancreatic cancer. Cancer Sci..

[70] Uno M., Kokuryo T., Yokoyama Y., Senga T., Nagino M. (2016). α-bisabolol inhibits invasiveness and motility in pancreatic cancer through KISS1R activation. Anticancer Res..

[71] Blundell R. (2007). Acute Leukaemia. Int. J. Mol. Med. Adv. Sci..

[72] Cavalieri E., Rigo A., Bonifacio M., Carcereri de Prati A., Guardalben E., Bergamini C., Fato R., Pizzolo G., Suzuki H., Vinante F. (2011). Pro-apoptotic activity of α-Bisabolol in preclinical models of primary human acute leukemia cells. J. Transl. Med..

[73] Costarelli L., Malavolta M., Giacconi R., Cipriano C., Gasparini N., Tesei S., Pierpaoli S., Orlando F., Suzuki H., Perbellini L. (2010). In vivo effect of alpha-Bisabolol, a nontoxic sesquiterpene alcohol, on the induction of spontaneous mammary tumors in HER-2/neu transgenic mice. Oncol. Res..

[74] Murata Y., Kokuryo T., Yokoyama Y., Yamaguchi J., Miwa T., Shibuya M., Yamamoto Y., Nagino M. (2017). The anticancer effects of novel α-bisabolol derivatives against pancreatic cancer. Anticancer Res..

[75] Piochon M., Legault J., Gauthier C., Pichette A. (2009). Synthesis and cytotoxicity evaluation of natural alpha-Bisabolol beta-D-fucopyranoside and analogues. Phytochemistry.

[76] Da Silva A.P., Martini M.V., de Oliveira C.M., Cunha S., de Carvalho J.E., Ruiz A.L., da Silva C.C. (2010). Antitumor activity of (-)-alpha-Bisabolol-based thiosemicarbazones against human tumor cell lines. Eur. J. Med. Chem..

[77] Smith E. (2018). Advances in understanding nociception and neuropathic pain. J. Neurol..

[78] Melo L.T., Panchalingam V., Cherkas P., Campos A.R., Avivi-Arber L., Sessle B.J. (2019). (-)-α-Bisabolol reduces nociception and trigeminal central sensitisation in acute orofacial neuropathic pain induced by infraorbital nerve injury. Life Sci..

[79] Fontinele L.L., Heimfarth L., Pereira E.W.M., Rezende M.M., Lima N.T., Barbosa Gomes de Carvalho Y.M., Afonso de Moura Pires E., Guimarães A.G., Bezerra Carvalho M.T., de Souza Siqueira Barreto R. (2019). Anti-hyperalgesic effect of (-)-α-Bisabolol and (-)-α-Bisabolol /β-Cyclodextrin complex in a chronic inflammatory pain model is associated with reduced reactive gliosis and cytokine modulation. Neurochem. Int..

[80] Melo L.T., Duailibe M.A., Pessoa L.M., da Costa F.N., Vieira-Neto A.E., de Vasconcellos Abdon A.P., Campos A.R. (2017). (-)-α-Bisabolol reduces orofacial nociceptive behavior in rodents. Naunyn. Schmiedebergs Arch. Pharm..

[81] Leite Gde O., Fernandes C.N., de Menezes I.R., da Costa J.G., Campos A.R. (2012). Attenuation of visceral nociception by α-Bisabolol in mice: Investigation of mechanisms. Org. Med. Chem. Lett..

[82] Alves Ade M., Gonçalves J.C., Cruz J.S., Araújo D.A. (2010). Evaluation of the sesquiterpene (-)-alpha-Bisabolol as a novel peripheral nervous blocker. Neurosci. Lett..

[83] Rocha N.F., Rios E.R., Carvalho A.M., Cerqueira G.S., Lopes Ade A., Leal L.K., Dias M.L., de Sousa D.P., de Sousa F.C. (2011). Anti-nociceptive and anti-inflammatory activities of (-)-α-Bisabolol in rodents. Naunyn. Schmiedebergs Arch. Pharm..

[84] Leite Gde O., Leite L.H., Sampaio Rde S., Araruna M.K., de Menezes I.R., da Costa J.G., Campos A.R. (2011). (-)-α-Bisabolol attenuates visceral nociception and inflammation in mice. Fitoterapia.

[85] Teixeira G.F., Costa F.N., Campos A.R. (2017). Corneal antinociceptive effect of (-)-α-Bisabolol. Pharm. Biol..

[86] Ortiz M.I., Cariño-Cortés R., Ponce-Monter H.A., Castañeda-Hernández G., Chávez-Piña A.E. (2018). Pharmacological interaction of α-Bisabolol and diclofenac on nociception, inflammation, and gastric integrity in rats. Drug Dev. Res..

[87] Teixeira G.F.D., Vieira-Neto A.E., da Costa F.N., ARA E.S., Campos A.R. (2017). Antinociceptive effect of (-)-α-Bisabolol in nanocapsules. Biomed. Pharm..

[88] Amora-Silva B.F., Ribeiro S.C., Vieira C.L., Mendes F.R., Vieira-Neto A.E., Abdon A.P.V., Costa F.N., Campos A.R. (2019). Clinical efficacy of new α-Bisabolol mouthwashes in postoperative complications of maxillofacial surgeries: A randomized, controlled, triple-blind clinical trial. Clin. Oral Investig..

[89] Heusch G. (2020). Myocardial ischaemia-reperfusion injury and cardioprotection in perspective. Nat. Rev. Cardiol..

[90] Lu L., Liu M., Sun R., Zheng Y., Zhang P. (2015). Myocardial infarction: Symptoms and treatments. Cell Biochem. Biophys..

[91] Nagoor Meeran M.F., Azimullah S., Laham F., Tariq S., Goyal S.N., Adeghate E., Ojha S. (2020). α-Bisabolol protects against β-adrenergic agonist-induced myocardial infarction in rats by attenuating inflammation, lysosomal dysfunction, NLRP3 inflammasome activation and modulating autophagic flux. Food Funct..

[92] Nagoor Meeran M.F., Laham F., Azimullah S., Tariq S., Ojha S. (2019). α-Bisabolol abrogates isoproterenol-induced myocardial infarction by inhibiting mitochondrial dysfunction and intrinsic pathway of apoptosis in rats. Mol. Cell Biochem..

[93] Meeran M.F.N., Laham F., Al-Taee H., Azimullah S., Ojha S. (2018). Protective effects of α-Bisabolol on altered hemodynamics, lipid peroxidation, and nonenzymatic antioxidants in isoproterenol-induced myocardial infarction: In vivo and in vitro evidences. J. Biochem. Mol. Toxicol..

[94] Hamedi A., Sakhteman A., Moheimani S.M. (2021). An in silico approach towards investigation of possible effects of essential oils constituents on receptors involved in cardiovascular diseases (CVD) and associated risk factors (Diabetes Mellitus and Hyperlipidemia). Cardiovasc. Hematol. Agents Med. Chem..

[95] Amerikova M., Pencheva I., Maslarska V., Bozhanov S., Tachkov K. (2019). Antimicrobial activity, mechanism of action, and methods for stabilisation of defensins as new therapeutic agents. Biotechnol. Biotechnol. Equip..

[96] Amenu D. (2014). Antimicrobial activity of medicinal plant extracts and their synergistic effect on some selected pathogens. Am. J. Ethnomed..

[97] Oliveira F.S., Freitas T.S., Cruz R.P.D., Costa M.D.S., Pereira R.L.S., Quintans-Júnior L.J., Andrade T.A., Menezes P.D.P., Sousa B.M.H., Nunes P.S. (2017). Evaluation of the antibacterial and modulatory potential of α-Bisabolol, β-cyclodextrin and α-Bisabolol /β-cyclodextrin complex. Biomed. Pharm..

[98] Pereira da Cruz R., Sampaio de Freitas T., Socorro Costa M.d., Lucas Dos Santos A.T., Ferreira Campina F., Pereira R.L.S., Bezerra J.W.A., Quintans-Júnior L.J., De Souza Araújo A.A., Júnior J.P.D.S. (2020). Effect of α-Bisabolol and its β-cyclodextrin complex as TetK and NorA efflux pump inhibitors in staphylococcus aureus strains. Antibiotics.

[99] Forrer M., Kulik E.M., Filippi A., Waltimo T. (2013). The antimicrobial activity of alpha-Bisabolol and tea tree oil against Solobacterium moorei, a Gram-positive bacterium associated with halitosis. Arch. Oral. Biol..

[100] Brehm-Stecher B.F., Johnson E.A. (2003). Sensitization of *Staphylococcus aureus* and *Escherichia coli* to antibiotics by the sesquiterpenoids nerolidol, farnesol, Bisabolol, and apritone. Antimicrob. Agents Chemother..

[101] Elgharbawy A., Samsudin N., Hashim Y., Salleh H., Santhanam J., Ben belgacem F. (2020). Phytochemicals with antifungal properties: Cure from nature. Int. J. Eng. Computat..

[102] Jahanshiri Z., Shams-Ghahfarokhi M., Asghari-Paskiabi F., Saghiri R., Razzaghi-Abyaneh M. (2017). α-Bisabolol inhibits Aspergillus fumigatus Af239 growth via affecting microsomal ∆(24)-sterol methyltransferase as a crucial enzyme in ergosterol biosynthesis pathway. World J. Microbiol. Biotechnol..

[103] Romagnoli C., Baldisserotto A., Malisardi G., Vicentini C.B., Mares D., Andreotti E., Vertuani S., Manfredini S. (2015). A multi-target approach toward the development of novel candidates for antidermatophytic activity: Ultrastructural evidence on α-bisabolol -treated microsporum gypseum. Molecules.

[104] De Medeiros C.A.C., Pinto Â.V., de Oliveira J.C., Silva G.S., Arrua J.M.M., Lima I.O., Pereira F.O. (2021). Evaluating the antifungal activity of α-bisabolol in association with NaCl on Fusarium oxysporum in maize grains. Curr. Microbiol..

[105] De Menezes R., Sampaio T.L., Lima D.B., Sousa P.L., de Azevedo I.E.P., Magalhães E.P., Tessarolo L.D., Marinho M.M., Dos Santos R.P., Martins A.M.C. (2019). Antiparasitic effect of (-)-α-Bisabolol against Trypanosoma cruzi Y strain forms. Diagn. Microbiol. Infect. Dis..

[106] Morales-Yuste M., Morillas-Márquez F., Martín-Sánchez J., Valero-López A., Navarro-Moll M.C. (2010). Activity of (-)alpha-Bisabolol against Leishmania infantum promastigotes. Phytomedicine.

[107] Hajaji S., Sifaoui I., López-Arencibia A., Reyes-Batlle M., Jiménez I.A., Bazzocchi I.L., Valladares B., Akkari H., Lorenzo-Morales J., Piñero J.E. (2018). Leishmanicidal activity of α-Bisabolol from Tunisian chamomile essential oil. Parasitol. Res..

[108] Rottini M.M., Amaral A.C., Ferreira J.L., Silva J.R., Taniwaki N.N., Souza Cda S., d’Escoffier L.N., Almeida-Souza F., Hardoim Dde J., Gonçalves da Costa S.C. (2015). In vitro evaluation of (-)α-Bisabolol as a promising agent against *Leishmania amazonensis*. Exp. Parasitol..

[109] Corpas-López V., Merino-Espinosa G., Díaz-Sáez V., Morillas-Márquez F., Navarro-Moll M.C., Martín-Sánchez J. (2016). The sesquiterpene (-)-α-Bisabolol is active against the causative agents of Old World cutaneous leishmaniasis through the induction of mitochondrial-dependent apoptosis. Apoptosis.

[110] Corpas-López V., Morillas-Márquez F., Navarro-Moll M.C., Merino-Espinosa G., Díaz-Sáez V., Martín-Sánchez J. (2015). (-)-α-Bisabolol, a Promising oral compound for the treatment of visceral leishmaniasis. J. Nat. Prod..

[111] Corpas-López V., Merino-Espinosa G., Acedo-Sánchez C., Díaz-Sáez V., Navarro-Moll M.C., Morillas-Márquez F., Martín-Sánchez J. (2018). Effectiveness of the sesquiterpene (-)-α-Bisabolol in dogs with naturally acquired canine leishmaniosis: An exploratory clinical trial. Vet. Res. Commun..

[112] Corpas-López V., Merino-Espinosa G., López-Viota M., Gijón-Robles P., Morillas-Mancilla M.J., López-Viota J., Díaz-Sáez V., Morillas-Márquez F., Navarro Moll M.C., Martín-Sánchez J. (2016). Topical treatment of *Leishmania tropica* Infection using (-)-α-Bisabolol ointment in a hamster model: Effectiveness and safety assessment. J. Nat. Prod..

[113] Fornai M., Antonioli L., Colucci R., Tuccori M., Blandizzi C. (2011). Pathophysiology of gastric ulcer development and healing: Molecular mechanisms and novel therapeutic options. Peptic Ulcer Disease.

[114] Bezerra S.B., Leal L.K., Nogueira N.A., Campos A.R. (2009). Bisabolol-induced gastroprotection against acute gastric lesions: Role of prostaglandins, nitric oxide, and KATP+ channels. J. Med. Food.

[115] Moura Rocha N.F., Venâncio E.T., Moura B.A., Gomes Silva M.I., Aquino Neto M.R., Vasconcelos Rios E.R., de Sousa D.P., Mendes Vasconcelos S.M., de França Fonteles M.M., de Sousa F.C. (2010). Gastroprotection of (-)-alpha-Bisabolol on acute gastric mucosal lesions in mice: The possible involved pharmacological mechanisms. Fundam. Clin. Pharm..

[116] Rocha N.F., Oliveira G.V., Araújo F.Y., Rios E.R., Carvalho A.M., Vasconcelos L.F., Macêdo D.S., Soares P.M., Sousa D.P., Sousa F.C. (2011). (-)-α-Bisabolol -induced gastroprotection is associated with reduction in lipid peroxidation, superoxide dismutase activity and neutrophil migration. Eur. J. Pharm. Sci..

[117] Soares R.O.S., Losada D.M., Jordani M.C., Évora P., Castro-e-Silva O. (2019). Ischemia/Reperfusion injury revisited: An overview of the latest pharmacological strategies. Int. J. Mol. Sci..

[118] Sampaio T.L., Menezes R.R., da Costa M.F., Meneses G.C., Arrieta M.C., Chaves Filho A.J., de Morais G.B., Libório A.B., Alves R.S., Evangelista J.S. (2016). Nephroprotective effects of (-)-α-Bisabolol against ischemic-reperfusion acute kidney injury. Phytomedicine.

[119] Sampaio T.L., Menezes R., Lima D.B., Costa Silva R.A., de Azevedo I.E.P., Magalhães E.P., Marinho M.M., Dos Santos R.P., Martins A.M.C. (2019). Involvement of NADPH-oxidase enzyme in the nephroprotective effect of (-)-α-Bisabolol on HK2 cells exposed to ischemia - reoxygenation. Eur. J. Pharmacol..

[120] Roe K. (2021). An inflammation classification system using cytokine parameters. Scand. J. Immunol..

[121] Xu C., Sheng S., Dou H., Chen J., Zhou K., Lin Y., Yang H. (2020). α-Bisabolol suppresses the inflammatory response and ECM catabolism in advanced glycation end products-treated chondrocytes and attenuates murine osteoarthritis. Int. Immunopharmacol..

[122] D’Almeida A.P.L., Pacheco de Oliveira M.T., de Souza É.T., de Sá Coutinho D., Ciambarella B.T., Gomes C.R., Terroso T., Guterres S.S., Pohlmann A.R., Silva P.M. (2017). α-Bisabolol-loaded lipid-core nanocapsules reduce lipopolysaccharide-induced pulmonary inflammation in mice. Int. J. Nanomed..

[123] Muñoz-Pérez V.M., Ortiz M.I., Ponce-Monter H.A., Monter-Pérez V., Barragán-Ramírez G. (2018). Anti-inflammatory and utero-relaxant effect of α-Bisabolol on the pregnant human uterus. Korean J. Physiol. Pharm..

[124] Cavalcante H.A.O., Silva-Filho S.E., Wiirzler L.A.M., Cardia G.F.E., Uchida N.S., Silva-Comar F.M.S., Bersani-Amado C.A., Cuman R.K.N. (2020). Effect of (-)-α-Bisabolol on the inflammatory response in systemic infection experimental model in C57BL/6 mice. Inflammation.

[125] Kim S., Jung E., Kim J.H., Park Y.H., Lee J., Park D. (2011). Inhibitory effects of (-)-α-Bisabolol on LPS-induced inflammatory response in RAW264.7 macrophages. Food Chem. Toxicol..

[126] Maurya A.K., Singh M., Dubey V., Srivastava S., Luqman S., Bawankule D.U. (2014). α-(-)-Bisabolol reduces pro-inflammatory cytokine production and ameliorates skin inflammation. Curr. Pharm. Biotechnol..

[127] Jat D., Nahar M. (2017). Oxidative stress and antioxidants: An overview. IJARR Int. J. Adv. Res. Rev..

[128] Zhang Y.-J., Gan R.-Y., Li S., Zhou Y., Li A.-N., Xu D.-P., Li H.-B. (2015). Antioxidant phytochemicals for the prevention and treatment of chronic diseases. Molecules.

[129] Braga P.C., Dal Sasso M., Fonti E., Culici M. (2009). Antioxidant activity of Bisabolol: Inhibitory effects on chemiluminescence of human neutrophil bursts and cell-free systems. Pharmacology.

[130] Ren G., Xue P., Sun X., Zhao G. (2018). Determination of the volatile and polyphenol constituents and the antimicrobial, antioxidant, and tyrosinase inhibitory activities of the bioactive compounds from the by-product of Rosa rugosa Thunb. var. plena Regal tea. BMC Complement Altern. Med..

[131] Thakur M., Singh K., Khedkar R., Prakash B. (2020). 11—Phytochemicals: Extraction process, safety assessment, toxicological evaluations, and regulatory issues. Functional and Preservative Properties of Phytochemicals.

[132] Api A.M., Belsito D., Biserta S., Botelho D., Bruze M., Burton G.A., Buschmann J., Cancellieri M.A., Dagli M.L., Date M. (2020). RIFM fragrance ingredient safety assessment, α-Bisabolol, CAS registry number 515-69-5. Food Chem. Toxicol..

[133] Bhatia S.P., McGinty D., Letizia C.S., Api A.M. (2008). Fragrance material review on alpha-Bisabolol. Food Chem. Toxicol..

[134] Gomes-Carneiro M.R., Dias D.M., De-Oliveira A.C., Paumgartten F.J. (2005). Evaluation of mutagenic and antimutagenic activities of alpha-Bisabolol in the Salmonella/microsome assay. Mutat. Res..

[135] Hernandez-Ceruelos A., Sánchez-Gutiérrez M., Mojica-Villegas M., Chamorro G. (2007). Chemoprotection of fertility by chamomile essential oil over the toxic effect of. Toxicol. Lett..

